# A novel codon-based de Bruijn graph algorithm for gene construction from unassembled transcriptomes

**DOI:** 10.1186/s13059-016-1094-x

**Published:** 2016-11-17

**Authors:** Gongxin Peng, Peifeng Ji, Fangqing Zhao

**Affiliations:** 1 Computational Genomics Lab, Beijing Institutes of Life Science, Chinese Academy of Sciences, Beijing, China; 2 University of Chinese Academy of Sciences, Beijing, China

**Keywords:** *de Bruijn* graph, Gene prediction, Phylogenomics, Transcriptome

## Abstract

Most gene prediction methods detect coding sequences from transcriptome assemblies in the absence of closely related reference genomes. Such methods are of limited application due to high transcript fragmentation and extensive assembly errors, which may lead to redundant or false coding sequence predictions. We present inGAP-CDG, which can construct full-length and non-redundant coding sequences from unassembled transcriptomes by using a codon-based de Bruijn graph to simplify the assembly process and a machine learning-based approach to filter false positives. Compared with other methods, inGAP-CDG exhibits a significant increase in predicted coding sequence length and robustness to sequencing errors and varied read length.

**Electronic supplementary material:**

The online version of this article (doi:10.1186/s13059-016-1094-x) contains supplementary material, which is available to authorized users.

## Background

Gene prediction provides basic functional information for understanding the genome sequence of a species and has become a crucial component of many frameworks used in genomic studies. With the rapid development of sequencing technology, transcriptome sequencing has become an efficient and cost-effective method for generating vast sequencing data for the prediction of genes for phylogenomic studies. Phylogenetically, orthologous genes are defined as genes descended from the sequence of a common ancestor through speciation [1]. The reliable identification of orthologous genes derived from high-quality coding sequences (CDSs) is critical for phylogenetic tree construction, an important component of many phylogenomic and functional studies. However, orthology inference is especially challenging for datasets reliant on transcriptomes containing misassemblies and partial or missing genes [2]. In particular, in eukaryotic transcriptomes, many genes have multiple isoforms, which may result in monophyletic or paraphyletic tips on the phylogenetic tree. For example, a widely used transcriptome assembler, Trinity [3], usually assembles many isoform groups (a subcomponent) for a given gene locus.

For species with reference genomes, functional genes are usually predicted using homology-based methods, which can identify genes by aligning target sequences to the original genes of closely related species. However, the reference database only represents a small fraction of existing species, limiting such methods to the sequences collected. Thus, gene prediction methods relying on known reference genomes limit our functional understanding of novel species. When related reference genomes are lacking, ab initio prediction methods utilizing assembled genomic sequences are inherently difficult due to the quality of training datasets [4–8]. Korf et al. found that in the absence of sufficient training data, GenScan [9] exhibited poor performance, with a sensitivity of 22.1% and a specificity of 20.0% for gene prediction in *Drosophila melanogaster* [7]. Alternatively, gene prediction can be performed based on de novo transcriptome assembly, which can considerably reduce the size of the dataset and increase the functional information obtained compared with genome sequencing. However, these methods are significantly limited by the quality of de novo transcriptome assembly, which is sensitive to sequencing errors, repetitive sequences in different genes, and the overlap of transcripts encoded by adjacent loci [3]. Hence, a typical transcriptome assembly may result in a large set of fragmented, redundant, and error-containing transcripts. For instance, an RNA-sequencing (RNA-seq) Genome Annotation Assessment Project (RGASP) competition study revealed that the highest accuracy of transcript assembly was only 48% for the RNA-seq reads of three transcriptome datasets [10]. Therefore, orthologous gene datasets derived from assembled transcripts are usually incomplete, fragmented, and redundant and often contain errors and isoforms that fundamentally skew the underlying assumptions of orthology inference in phylogenomic analyses.

**Fig. 1:**
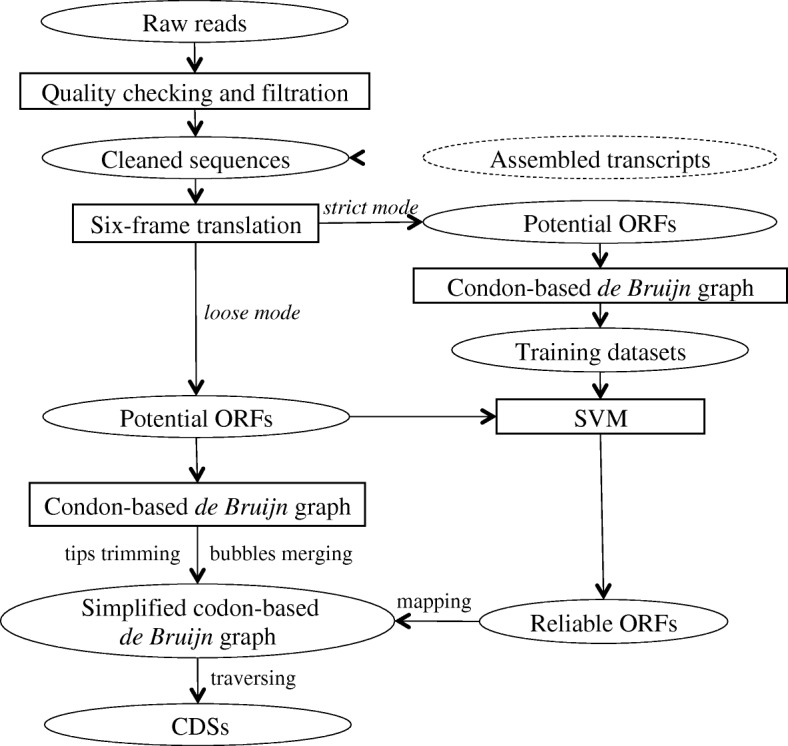
The workflow of the inGAP-CDG algorithm. The inGAP-CDG algorithm mainly includes four steps: six-frame translation, SVM filtration, codon-based de Bruijn graph construction, and traversal. First, input sequences (reads, merged reads, or assembled transcripts) are translated into potential ORFs. Next, inGAP-CDG collects highly reliable ORFs (rORFs) with strict parameters using a combination of SVM and a codon-based de Bruijn graph. Lastly, inGAP-CDG builds a codon-based de Bruijn graph based on all of the predicted ORFs and generates full-length CDSs by traversing the graph under the guidance of rORFs

To overcome this difficulty and to increase the utility of transcriptome datasets, we developed inGAP-CDG, an algorithm that can perform gene construction from unassembled transcriptomes. Compared with previous approaches, inGAP-CDG predicts open reading frames (ORFs) directly from unassembled reads, exploits a supervised support vector machine (SVM) to filter false-positive ORFs, and employs a novel codon-based de Bruijn graph to assemble cleaned ORFs into full-length CDSs (Fig. 1). Using both simulated and real datasets, we demonstrated that inGAP-CDG can significantly improve the length and precision of gene recognition. inGAP-CDG is implemented in C++ and the source code is freely available together with full documentation at https://sourceforge.net/projects/ingap-cdg.

## Results

### Codon-based de Bruijn graph versus traditional de Bruijn graph

**Fig. 2:**
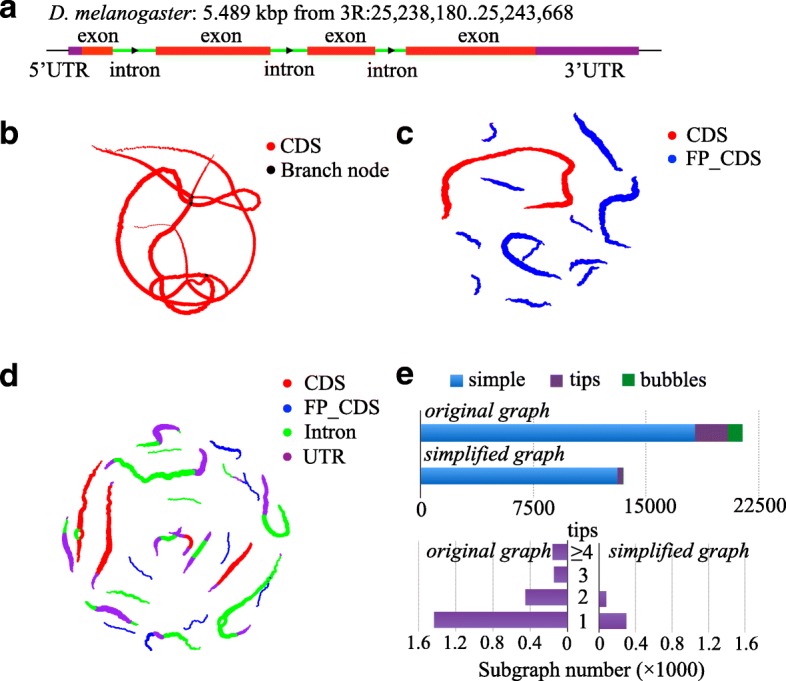
Comparison between the traditional de Bruijn graph and the codon-based de Bruijn graph. **a** The basic information of FBgn0039298 gene in *D. melanogaster* used for generating the subfigures (**b**)–(**d**). The elements are marked with different *colors* to highlight the gene structure (UTR: *purple*, intron: *green*, exon: *red*). **b** Traditional de Bruijn graph based on simulated DNA-seq reads on CDS regions. **c** Codon-based de Bruijn graph based on simulated RNA-seq reads. Each *dot* denotes a kmer node. The nodes belonging to CDS are shown in *red*. The false CDSs translated from the wrong frames of the gene are shown in *blue*. **d** Codon-based de Bruijn graph based on simulated DNA-seq reads on the whole gene. The nodes belonging to CDSs are shown in *red*. The false CDSs translated from the wrong frames of the gene are shown in *blue*. The false CDSs translated from introns and UTRs are shown in *green* and *purple*, respectively. **e** Comparison between the two codon-based de Bruijn graphs before and after tips trimming and bubble merging using a real RNA-seq dataset (ERR188040). “Simple” indicates subgraphs without tips and bubbles; “tips” indicates subgraphs containing tips; “bubbles” indicates subgraphs containing bubbles and/or tips

As shown in Additional file 1: Supplementary Methods, we have offered mathematical proof demonstrating that the codon-based de Bruijn graph exhibits a substantial advantage over the traditional de Bruijn graph due to a decrease in graph nodes and edges. To further demonstrate this reduction and to quantify the difference between codon-based and traditional graphs, we used both simulated and real datasets to compare their properties. We generated three chr3 Consensus Coding Sequences (CCDSs) annotated datasets for human, mouse, and fruit fly, respectively. For each dataset, both a traditional graph and a codon-based de Bruijn graph were constructed. The number of nodes and edges in each graph was calculated and compared. As shown in Additional file 1: Table S1, the total number of nodes and edges in the constructed codon-based de Bruijn graph was approximately one-third that of the traditional graph, which was consistent with the theoretical result because there were no sequencing errors or false frame translations in the CCDS. We further compared results for three real datasets (ERR188040, ERR1161592, and SRR1045067) (Additional file 1: Table S2) and achieved similar results. The number of nodes and edges was approximately half that of the traditional graph, indicating that sequencing errors and false frame translations increased the complexity of the codon-based de Bruijn graph. To obtain a more intuitive and comprehensive understanding of the codon-based de Bruijn graph, the gene FBgn0039298 was taken as an example. Using all of the exons of this gene, traditional (Fig. 2a) and codon-based de Bruijn graphs (Fig. 2b) were constructed. As shown in Fig. 2, the codon-based de Bruijn graph assembled all exons of this gene into one simple path (Fig. 2c) and exhibited considerably decreased complexity compared with the traditional graph (Fig. 2b). It should be noted that the codon-based de Bruijn graph also included a false CDS that did not overlap with the true CDS and thus could be easily discarded in downstream cleaning steps. This reduced complexity was even more evident when using all elements of the gene to construct the codon-based de Bruijn graph. As shown in Fig. 2d, the graph was composed of 22 components and most of the components contained only one path. Moreover, the four exons were assembled into four separate components, each of which contained few overlaps with other elements.

The codon-based de Bruijn graph facilitates the assembly not only by decreasing the number of nodes and edges but also by reducing the topological complexity. To demonstrate this point, we used the codon-based de Bruijn graph after simplification as a benchmark. We first classified the components into three types: simple, tip-containing, and bubble-containing subgraphs. Then, the number of each type of subgraph was calculated for the graphs before and after simplification. As shown in Fig. 2e, the most dominant components in both graphs were simple paths. It is important to note that even for the graph containing sequencing errors and frameshifting (Fig. 2e), 85% of the structures were simple subgraphs. Moreover, most tip-containing subgraphs had only one tip, which could easily be trimmed into simple subgraphs. Therefore, after performing graph simplification, the number of simple subgraphs increased from 85% to 96%, indicating the outstanding performance of codon-based de Bruijn graphs for CDS construction and recognition.

### SVM filtration

**Fig. 3:**
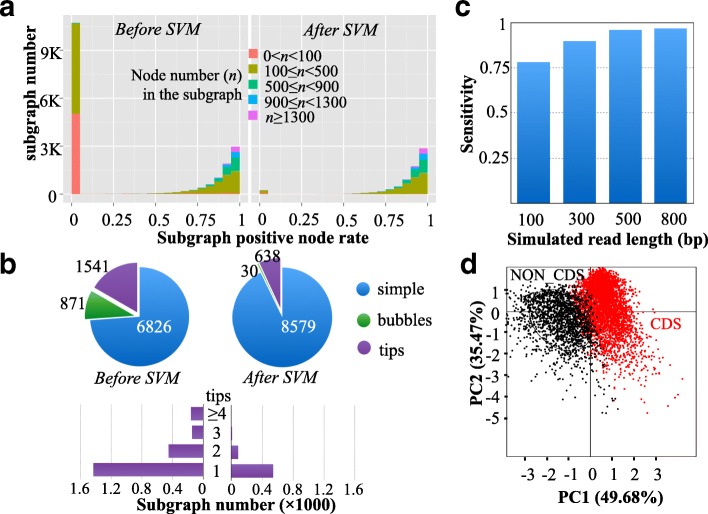
Performance evaluation of SVM filtration. **a** Performance of SVM on filtering false-positive ORFs. The subgraph number and the ratio of false-positive nodes in each subgraph were calculated before and after SVM filtration. **b** Comparison between the two codon-based de Bruijn graphs before and after SVM filtration. **c** Sensitivity of true ORF recovery after SVM filtration using four simulated datasets with different read lengths (100, 300, 500, and 800 bp). **d** Classification results of SVM filtration through reducing the high-dimensional feature vectors by principal component analysis. *Red dots* indicate true ORFs belonging to CDSs, whereas *black dots* represent false ORFs derived from non-CDSs

To evaluate the efficiency of SVM in removing false-positive ORFs, we utilized three simulated RNA-seq datasets based on the sequences of human chr3, chr19, and chr20. First, positive and negative datasets were prepared to test SVM filtration. For each chromosome, the simulated reads were translated into ORFs, which were used as the test dataset. The CDSs of the chromosome were used as the positive dataset, while the sequences translated from the other five reading frames of each CDS were used as the negative dataset. Then, the sensitivity and specificity were calculated by aligning the predicted ORFs to the reference CDSs. As shown in Additional file 1: Figure S1A–C, SVM successfully recovered true ORFs with an average sensitivity of 90% and a specificity of 75%. We simulated four datasets with read lengths of 100, 300, 500, and 800 bp and the sensitivity after SVM filtration was calculated for each dataset. As shown in Fig. 3c, the sensitivity tended to improve with increasing read length. The primary factor responsible for this increase is likely to be sequence length because short sequences do not contain sufficient composition signals for discrimination.

To examine whether SVM filtration could reduce the complexity of the codon-based de Bruijn graph, we employed a real RNA-seq dataset (ERR188040) that was first assembled by Trinity and the resulting transcripts were translated into ORFs. Two codon-based de Bruijn graphs were constructed using the translated ORFs before and after SVM filtration, and the ratio of false-positive nodes in the components of each graph was calculated (Fig. 3a). Before SVM filtration, there were a large number of false-positive nodes on the graph, which were mainly present in small components (node number <500), indicating that false-positive nodes shared few overlaps with each other or with true-positive nodes. After filtration, SVM discarded almost all of these nodes, indicating its high efficiency. Moreover, the codon-based de Bruijn graph derived from SVM-filtered ORFs exhibited an approximately 63% decrease in the number of components compared with the graph derived before filtration (Fig. 3b). A subset of filtered translated ORFs was taken as an example to illustrate the SVM classification result. When reducing the high dimension of SVM prediction features by principal component analysis (PCA), two distinct clusters were observed (Fig. 3d).

We also tested the impact of SVM filtration on the assembled CDSs using sequenced reads (SRR1045067). The sequenced reads were first used to predict ORFs. inGAP-CDG yielded two CDS assemblies based on the predicted ORFs with or without SVM filtration. Contig length, redundancy, average fragment number per gene, sensitivity, and specificity of CDS recognition in the two assemblies were compared (Additional file 1: Figure S2). As expected, the CDS assembly with an SVM filtration step exhibited a significantly increased CDS length compared to that without filtration (Additional file 1: Figure S2A and S2B). In addition, the CDS assembly with SVM outperformed the CDS assembly without filtration in both sensitivity and specificity (Additional file 1: Figure S2D). Therefore, we concluded that SVM-based filtration not only reduced the complexity of the codon-based de Bruijn graph by filtering false-positive ORFs but also improved the performance of gene recognition from fragmented sequences.

### Gene prediction from assembled transcripts

Because most state-of-the-art gene prediction methods identify CDSs from assembly transcripts, we further tested the performance of inGAP-CDG on long transcripts. An RNA-seq dataset for human colon cancer cells (SRR1045067) was downloaded and assembled using the Trinity assembler. Both inGAP-CDG and TransDecoder were employed to predict CDSs from the resulting transcripts. As shown in Additional file 1: Figure S3A, inGAP-CDG exhibited considerably improved CDS length compared with TransDecoder. Moreover, N50, mean, and N90 length were approximately twice as long as those from TransDecoder (Additional file 1: Figure S3B). Subsequently, the predicted CDSs were aligned with the reference gene set and matched CDSs were extracted for comparison. We found that the average length of the matched CDSs predicted by inGAP-CDG was slightly shorter than that of the reference gene set, but it was significantly longer than that of TransDecoder. These nearly full-length CDSs will greatly facilitate downstream phylogenomic analyses and gene model construction. Next, the sensitivities and specificities of the two methods were compared and inGAP-CDG was found to exhibit higher specificity but slightly lower sensitivity (Additional file 1: Figure S3D). This decreased sensitivity is most likely due to SVM filtration, during which some true ORFs may be filtered out. Finally, we compared the redundancy of these CDSs and found that inGAP-CDS exhibited greatly decreased redundancy compared with TransDecoder (Additional file 1: Figure S3C and S3E).

### Performance comparison between inGAP-CDG and 11 other strategies

To evaluate the robustness of inGAP-CDG over different sequencing error rates, we simulated three datasets with error rates of 0.5%, 1%, and 2% and employed inGAP-CDG and 11 other pipelines to assemble and predict CDSs. The mean length, redundancy, sensitivity, and error rate were calculated and compared. As shown in Additional file 1: Figure S4, inGAP-CDG achieved the longest mean length and the lowest redundancy among all of the approaches for all three simulated datasets. Although inGAP-CDG exhibited a moderate level of sensitivity (approximately 90%) and base error rate (0.005–0.01%), it produced the smallest fluctuation in the face of different sequencing error rates.

To demonstrate the robustness of inGAP-CDG over different read lengths, we compared inGAP-CDG with the other 11 pipelines using three real datasets (ERR188040, ERR1161592, and SRR1045067) of different read lengths (75, 100, and 150 bp). As shown in Additional file 1: Figure S5, the results varied depending on the read length. First, inGAP-CDG had the largest mean CDS length among all of the methods. Unexpectedly, the mean CDS length decreased when the read length was increased from 75 bp to 150 bp. This decrease was observed not only for inGAP-CDG but also for the other methods and was most likely due to fact that the 150-bp reads contained more sequencing errors. Next, a comparison of sensitivity and specificity revealed that with an increase in read length, the overall sensitivities and specificities of all assemblies exhibited tendencies toward enhancement and reduction, respectively. In addition, inGAP-CDG achieved significantly higher specificity than all other methods. Notably, the specificities of these methods showed an increasing trend when increasing the read length from 75 bp to 150 bp. In contrast with this trend, inGAP-CDG exhibited a steady increase, demonstrating a high level of robustness for inGAP-CDG, which was achieved by discarding false translated ORFs resulting from sequencing errors and erroneous frameshifts. This robustness was also observed in the redundancy (Additional file 1: Figure S5C and S5D). Unlike other methods, which varied significantly when using different read lengths, inGAP-CDG exhibited only a slight fluctuation.

**Fig. 4:**
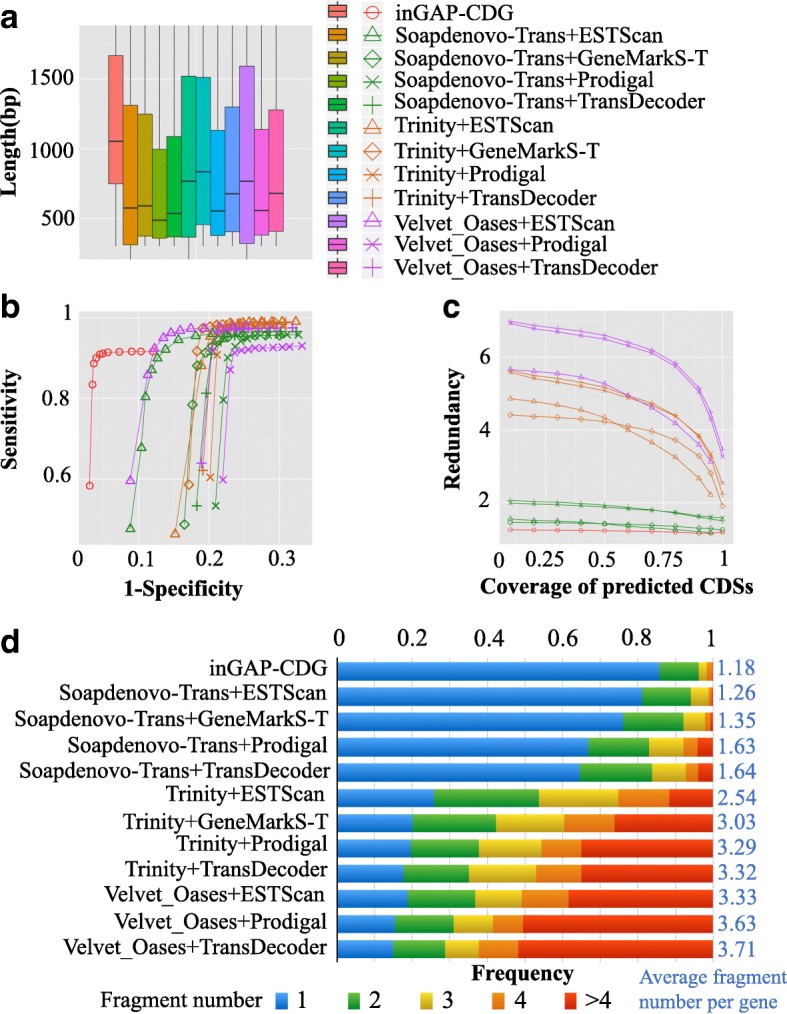
Performance comparison on CDS construction among inGAP-CDG and 11 other combined pipelines. **a** The length distribution of predicted CDSs by each method. **b** The ROC curve of each method. ROC, which captures the trade-offs between sensitivity and specificity, is determined by calculating the true-positive and false-positive rates. **c** The redundancy of predicted CDSs by each method. The redundancy is calculated by the number of aligned CDSs divided by the number of reference genes. **d** The evaluation of CDS fragmentation in each method. inGAP-CDG outperforms the other 11 pipelines, with an average of 1.18 fragments per gene

To benchmark the performance of inGAP-CDG, we compared this software with 11 other pipelines using a 150-bp paired-end RNA-seq dataset (SRR1045067) from *H. sapiens*. inGAP-CDG predicted CDSs directly from these unassembled reads. For each pipeline, these reads were first assembled and CDSs were then predicted. After CDS prediction, we compared the CDS length of each method and found that inGAP-CDG outperformed all other methods (Fig. 4a and b). Next, the predicted CDSs of each method were aligned with the reference gene set to compare sensitivity, specificity and redundancy. As shown in Fig. 4c and d, inGAP-CDS exhibited the highest specificity and the lowest redundancy. The succinate dehydrogenase (SDHA, accession ID: NM_004168) gene of NCBI database is an example of this performance (Additional file 1: Figure S6B). When aligning the predicted CDSs to this gene using the inGAP package [11, 12], different alignment profiles were observed among these methods. inGAP-CDG successfully assembled all of the reads of this gene into one CDS, and approximately 100% of the gene was covered by the predicted CDS. However, when using other methods, only a part of this gene was covered by the predicted CDSs and some of them included assembly chimeras. Moreover, the covered regions of this gene were aligned to multiple CDSs, a redundancy that was even more obvious in the CDSs predicted from Trinity-assembled transcripts. Similar findings were observed for the genes SET domain containing 2 (SETD, accession ID: XM_011533632), dipeptidyl peptidase 7 (DPP7, accession ID: NM_013379) and acyl-CoA dehydrogenase (ACADVL, accession ID: NM_000018), which are all found in the NCBI database (Additional file 1: Figure S6A, C, and D). Together, these comparisons indicate that inGAP-CDG is more reliable than other pipelines for full-length CDS prediction. We further benchmarked inGAP-CDG using three real RNA-seq datasets (SRR3332174, SRR3332175, and SRR3332176) from *D. melanogaster*. After gene construction, the mean length, sensitivity, redundancy, and chimera rate of inGAP-CDG were compared with those of the other pipelines. Although inGAP-CDG exhibited a moderate level of sensitivity, it outperformed all other pipelines in terms of the mean length, redundancy and chimera rate for these datasets (Additional file 1: Figure S7). It should be noted that inGAP-CDG could achieve an even longer mean CDS length and lower redundancy using the strict mode, at the expense of sensitivity (4–7% decrease).

### Application of inGAP-CDG to orthologous gene recognition

**Fig. 5:**
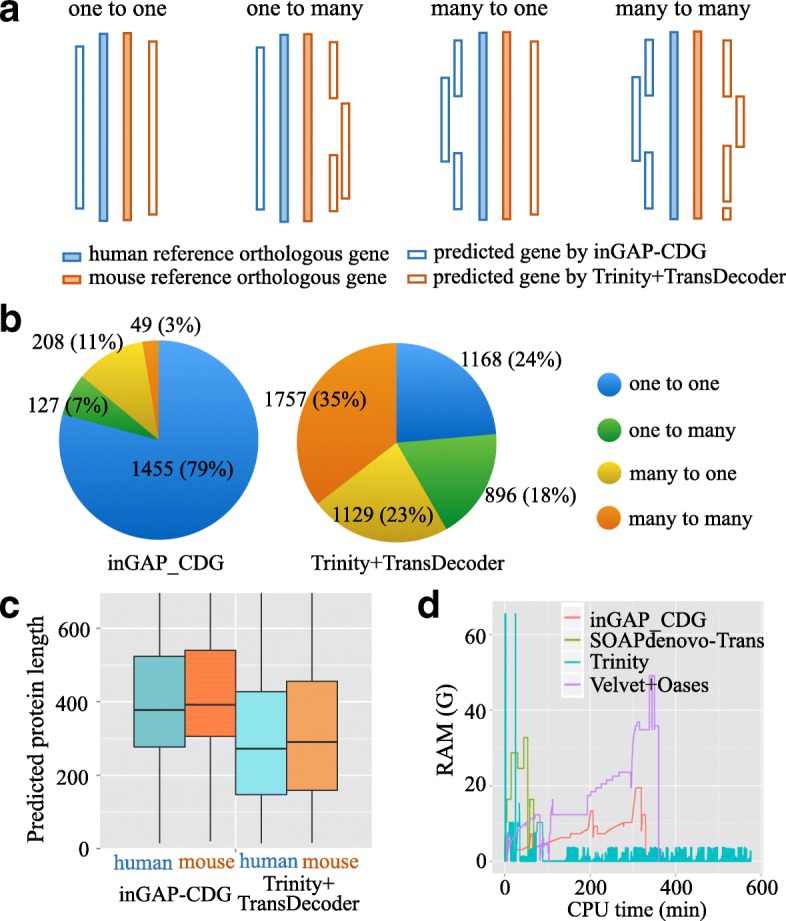
Application of inGAP-CDG on orthologous gene construction and its computational efficiency. RNA-seq datasets from human and mouse brain tissues were used to evaluate the performance of inGAP-CDG and Trinity + TransDecoder on detecting orthologous gene pairs. **a** Based on the alignments of predicted genes to reference orthologous gene groups, predicted orthologs can be classified into four groups. Only the ‘one-to-one’ group can be recognized as authentic orthologous pairs by classical orthology inference methods. **b** inGAP-CDG predicted more ‘one-to-one’ type orthologs than Trinity + TransDecoder. **c** The length distribution of predicted genes by the two methods. **d** Comparison of the running time and RAM usage among the four methods (inGAP-CDG, Trinity, Soapdenovo-Trans, Velvet + Oases) using the publicly available RNA-seq dataset (SRR1045067)

To assess the performance of inGAP-CDG in orthology detection, we compared inGAP-CDG with a pipeline that combined Trinity with TransDecoder using two real RNA-seq datasets from *H. sapiens* and *M. musculus* brain tissues. For each dataset, the proteins were predicted using these two strategies separately. After assembling the human dataset, inGAP-CDG yielded 14,638 ORFs with an average length of 419 codons, while the Trinity + TransDecoder pipeline produced 88,184 ORFs with an average length of 274 codons. For the mouse dataset, inGAP-CDG and the Trinity + TransDecoder pipeline generated 10,260 and 65,862 ORFs with average lengths of 481 and 304 codons, respectively. Subsequently, we retrieved the one-to-one orthologous gene pairs between *H. sapiens* and *M. musculus* from the OrthoMCL database into two reference gene sets according to their original species. For each species, the predicted proteins of these two strategies were aligned to the respective reference gene set. As a result, 3296 and 3258 proteins predicted by inGAP-CDG were aligned to the human and mouse reference gene sets, respectively, while 7219 and 7227 proteins predicted by Trinity + TransDecoder were aligned to the human and mouse reference gene sets, respectively. Finally, for each strategy, the numbers of aligned predicted proteins of two species for each paired orthologous gene were classified into four types (Fig. 5a), which represented the redundancy and completeness of each assembly and the number of predicted proteins that could be used for phylogenetic tree construction.

As shown in Fig. 5b, the most dominant type of proteins assembled in inGAP-CDG was one-to-one (79% of the aligned predicted proteins) and the smallest type was many-to-many, comprising only 3%. In contrast, many-to-many was the largest type in the Trinity + TransDecoder pipeline (35%), followed by one-to-one (24%), many-to-one (23%), and one-to-many (18%). The low fluctuation among these types indicated that this pipeline produced redundant and incomplete genes. Moreover, inGAP-CDG outperformed this pipeline by generating more one-to-one type and fewer fragmented orthologous genes, demonstrating its predominant advantage in phylogenomic studies. We also surveyed the length distribution of aligned translated proteins (Fig. 5c) and found that inGAP-CDG exhibited an increased length compared with the combined pipeline. Such increased length will greatly facilitate the completeness and accuracy of phylogenetic trees.

### inGAP-CDG running time and memory usage

inGAP-CDG is implemented in C++ as a standalone program. We have tested it successfully on Mac OS X EI Capitan (10.11) and Linux (Red Hat 6.3 and Ubuntu 16.04) systems. To systematically evaluate the efficiency of inGAP-CDG, the running time and RAM usage were compared with the four other gene prediction pipelines using a publicly available RNA-seq dataset (SRR1045067). Because the running time and memory usage of ESTScan, TransDecoder, Prodigal, and GeneMarkS-T were negligible, these tools were not included in this comparison. We ran all four programs on a node with 2.13 GHz Intel Xeon processors using eight CPUs on the Linux (Red Hat 6.3) system. As shown in Fig. 5d, among all of the programs, inGAP-CDG had the lowest peak RAM, which was approximately one-third of that of Trinity. In addition, inGAP-CDG was faster than Trinity and Velvet_Oases but slower than SOAPdenovo-Trans. For the dataset SRR1045067, consisting of 4.8G bases, inGAP-CDG took approximately 350 min to construct CDSs from raw reads. In detail, six-frame translation, SVM filtration, codon-based de Bruijn graph construction, and traversal took approximately 65, 135, 120, and 30 min, respectively.

## Discussion

Transcript-based gene prediction methods exhibit increased accuracy compared with ab initio methods, which require high-quality training datasets to ensure reliability. However, the limitations of current transcriptome assemblers have precluded these methods from generating high-quality and non-redundant CDSs. To address this challenge, we present a novel tool, inGAP-CDG, for the effective construction of full-length and non-redundant CDSs from unassembled transcriptomes. By introducing the newly developed codon-based de Bruijn graph to simplify the assembly process and SVM to filter false-positives, inGAP-CDG can predict full-length CDSs at a low level of redundancy and with a low false-positive rate.

Transcriptome sequencing is an efficient and cost-effective route for generating vast sequence collections representing expressed genes. This technology provides a valuable starting point for phylogenomic analysis in non-model organisms for which genomic sequence information is not yet available [13, 14], especially when whole-genome sequencing efforts are cost-prohibitive and time-prohibitive. Several gene recognition methods [15–17] that integrate de novo assembly and gene prediction have been proposed. However, these methods result in fragmented and redundant CDSs. These properties will lead to incomplete or false orthologous genes and thus generate low-quality and incongruent phylogenetic trees. However, inGAP-CDG addresses this challenge by generating more one-to-one orthologous genes and improving gene completeness for phylogenomic studies.

We have demonstrated that inGAP-CDG exhibited a great advantage over all currently available transcriptome-based gene prediction methods. The primary factor underlying this advantage is the implementation of a codon-based de Bruijn graph, which contains a considerably decreased number of nodes and edges (by approximately 60%) compared with the traditional graph. Moreover, most of the structures in the codon-based de Bruijn graph are simple components, indicating a low level of topological complexity. Collectively, these features allow for decreased complexity and redundancy in gene prediction. Similar to the codon-based de Bruijn graph, Youngik et al. employed an amino-acid alphabet-based de Bruijn graph to simplify the traditional de Bruijn graph and reconstructed protein sequences from next-generation sequencing (NGS) metagenomic data [18]. Because each character of the amino acid alphabet-based de Bruijn graph may be one of 20 amino acids (i.e. fivefold larger than the number of nucleotides), this type of graph contains an increased number of nodes and edges while exhibiting decreased topological complexity compared with the codon-based de Bruijn graph. Moreover, the outputted sequences of the amino acid alphabet-based de Bruijn graph are short peptides, which masks the original nucleic acid sequence. In addition to generating a codon-based de Bruijn graph, SVM filtration is an essential step in the inGAP-CDG algorithm. SVM discards false-positive CDSs generated by six-frame translation and yields reliable ORFs to serve as landmarks, which can dictate traversal. This filtration allows significantly increased specificity compared with other methods. Furthermore, inGAP-CDG directly recognizes CDSs from unassembled reads and consequently avoids the influence of uneven sequencing depth on assembly, which may enhance the CDS length.

Compared with other methods, inGAP-CDG exhibited a slight loss of sensitivity (approximately 6.5%) in gene recognition from unassembled transcriptomic reads. The main reason for this loss is the implementation of SVM filtration in the algorithm. To ensure the accuracy of the result, inGAP-CDG used strict parameters in SVM to filter false-positive ORFs, which may sacrifice a small fraction of true-positive ORFs. Moreover, this filtration ensures the robustness of inGAP-CDG to sequencing errors. Further efforts could be made to improve the sensitivity of inGAP-CDG by taking into account more information (e.g. the coverage of kmers by mapping reads to the codon-based de Bruijn graph, as well as paired-end read linkage information). We believe that inGAP-CDG is an important addition to the toolbox used for phylogenomic studies and will greatly improve our capacity to explore the functional potential of novel species.

## Conclusions

This study presents a novel algorithm, inGAP-CDG, for effective gene construction from unassembled transcriptomes. The main advantage of inGAP-CDG is that it combines a newly developed codon-based de Bruijn graph to simplify the assembly process and a machine learning based approach to filter false positives. Compared with traditional de Bruijn graph, the codon-based de Bruijn graph exhibits significantly decreased number of nodes and edges (by approximate 60%), as well as a considerable low level of topological complexity. These features of the codon-based de Bruijn graph allow decreased complexity and redundancy in gene prediction. Through extensive evaluation on both simulated and real datasets, as well as comparisons with alternative methods, we demonstrate that inGAP-CDG has an excellent and unbiased performance on gene reconstruction.

## Methods

### Overview of inGAP-CDG

The inGAP-CDG algorithm is a four-step process that includes six-frame translation, SVM filtration, codon-based de Bruijn graph construction, and traversal (Fig. 1). First, input sequences (e.g. reads or transcriptomic assemblies) are translated into potential ORFs. Due to sequencing and frame translation errors, a significant number of predicted ORFs are false positives. Next, inGAP-CDG employs a combination of SVM and codon-based de Bruijn graphing to obtain a set of highly reliable ORFs (rORFs) to serve as landmarks in the next step. Finally, because short predicted ORFs are occasionally discarded by SVM [19], inGAP-CDG builds a codon-based de Bruijn graph based on all predicted ORFs and generates full-length CDSs by traversing the graph under the guidance of rORFs (Additional file 1: Figure S8).

### Codon-based de Bruijn graph construction

The input sequences are split into kmers, with a sliding window of k (default of 27 bp) and a step size of 3 bp. Then, the codon-based de Bruijn graph is built using the resulting kmers. We define the directed de Bruijn graph as a codon-based graph G = (V, E) that has a set of vertices *V* = {*v*_1_, *v*_2_, …, *v*_*n*_} and a set of edges *E* = {(*v*_1_, *v*_5_), (*v*_1_, *v*_3_), …, (*v*_*m*_, *v*_*k*_)|*v*_*m*_, *v*_*k*_ ∈ *V*} that are formed by pairs of k-3 bp overlapping kmers. The set of nodes is created by assigning each kmer *v* ∈ *V* to a unique vertex. A path from ${v}_1$ to ${v}_j$ through the contig graph consists of a sequence of nodes (e.g. (*v*_1_, *v*_7_, *v*_4_, *v*_*j*_)). The graph G_1_ = (V_1_, E_1_) is a subgraph of G = (V, E) if (1) V_1_ ⊆ V and (2) every edge of G_1_ is also an edge of G. The subgraph G_1_ = (V_1_, E_1_) is a connected component of G = (V, E) if G_1_ satisfies three conditions: (1) V_1_ ⊆ V; (2) every edge of G_1_ is also an edge of G; and (3) any two nodes in G_1_ are connected to each other by a path, and no paths can be found to connect the nodes between V_1_ and (V–V_1_). A head is defined as the style of arrowhead on the head node of an edge and a tail is the style of arrowhead on the tail node of an edge.

### Six-frame translation

Sequences for six-frame translation can be single-end reads, paired-end reads, or transcriptomic assemblies. If paired-end reads are provided and each pair exhibits overlaps (insert size < the summed length of a read pair), these reads are merged into long sequences using FLASH [20] software. Otherwise, these reads are treated as single-end reads. Then, for each inputted sequence, the positions of all of the stop codons are recorded. Then, all translated ORFs are collected and sorted by length. In the six-frame translation of the inGAP-CDG algorithm, we obtain two types of ORFs based on different parameters. The first type includes strict-translated ORFs that are the same length as the original sequence and do not contain a stop codon. The second type consists of loose-translated ORFs that cover at least 80% of the original sequence (Additional file 1: Figure S9).

### SVM filtration

inGAP-CDG employs SVM to train the prediction model and to recognize candidate ORFs in the test dataset. The positive and negative datasets used in SVM are prepared using a codon-based de Bruijn graph strategy. First, inGAP-CDG chooses all ORFs that contain no stop codon and have the same length as the original sequence. Second, a codon-based de Bruijn graph is built based on these ORFs, and contigs are generated by traversing the graph. Third, the long contigs (default > = 1 kb) are used as the positive dataset, whereas for each long contig, the predicted ORFs for the other five frames are treated as the negative dataset. Finally, SVM trains a classification model by taking the codon usage frequency of each sequence as a feature vector to generate a set of rORFs from the test dataset (Additional file 1: Figure S8).

### CDG traversal and coding sequence generation

Because some true ORFs are discarded during SVM filtration, a codon-based de Bruijn graph constructed from rORFs alone may result in fragmented CDSs. To solve this problem, we generated a more complete but noisy graph using the SVM test dataset (all ORFs translated via six-frame translation) and mapped rORFs to the graph to generate full-length CDSs. In detail, we split the ORFs in the test dataset into kmers and constructed a new codon-based de Bruijn graph. To simplify this graph, we executed two functions: “tips trimming” and “bubbles merging” (Additional file 1: Figure S10A–F). If a “tip,” which is defined as a chain of nodes disconnected on one end, is shorter than a given length (default: 2*kmer), it will be removed. A “bubble” is defined as several similar paths with the same start and end nodes on the graph. The paths in a bubble are compared, and the identity among these paths is calculated. If the identity reaches a cutoff of 95%, the longest path is reserved, and all other paths are discarded. The rORFs are then mapped to the simplified graph and the mapped kmers, referred to as landmarks, will be used for traversing the graph. Rather than traversing the entire graph, inGAP-CDG employs a depth-first searching algorithm, which starts at each landmark and searches against the graph to identify the path that connects two landmarks. Once all landmarks have been visited, the nodes along the searched path are assembled into a final CDS. Notably, inGAP-CDG can be performed under two different modes: default and strict. The strict mode is designed to obtain longer unigenes with a lower level of redundancy. Unless specified, inGAP-CDG is performed in the default mode.

### Design of simulation studies

The *D. melanogaster* gene FBgn0039298, downloaded from FLYBASE [21], was used to compare the traditional and codon-based de Bruijn graphs. This gene is 5489 bp in length and contains four exons and three introns. First, a set of 100-bp single-end DNA-seq reads with a sequencing depth of 30-fold was simulated based on this gene using the Wgsim program from the SAMtools package [22] and the dataset was used by inGAP-CDG to construct a codon-based de Bruijn graph. Next, a set of 100-bp single-end RNA-seq reads was simulated from the CDSs of this gene using FluxSimulator [23] to construct a traditional and a codon-based de Bruijn graph.

To test the feasibility of SVM filtration, three sets of 150-bp single-end RNA-seq reads were simulated from chr3, chr19, and chr20 of the reference human genome (hg19) using FluxSimulator with the default parameters. These simulated datasets were then subjected to SVM filtration as implemented in inGAP-CDG. After SVM filtration, the receiver operating characteristic (ROC) curve of each dataset was plotted in R using the LIBSVM packages (e1071). To test the effect of read length on the performance of inGAP-CDG, four RNA-seq datasets with read lengths of 100, 300, 500, and 800 were simulated from the CCDS of hg19.

To test the performance of inGAP-CDG using transcriptomic data with sequencing errors, three sets of 200-bp paired-end RNA-seq reads were simulated from chr3 of hg19 using RNASeqReadSimulator (https://github.com/davidliwei/RNASeqReadSimulator) with error rates of 0.5%, 1%, and 2%, respectively. Furthermore, the metrics of sensitivity, redundancy, mean length, and base error rate were used to assess the resulting CDS predictions.

### Real datasets

Three paired-end RNA-seq datasets generated from *Homo sapiens* were downloaded from the NCBI Sequence Read Archive (SRA) database [24] (accession numbers ERR188040, ERR1161592, and SRR1045067). These datasets contained 27.8, 24.4, and 19.2 million read pairs with read lengths of 75, 100, and 150 bp, respectively. Because the paired reads of the SRR1045067 dataset contained overlaps, we merged these reads into long reads using FLASH and obtained 13.4 million merged reads.

To verify the performance of inGAP-CDG on real datasets, three paired-end RNA-seq datasets from *D. melanogaster* (accession numbers: SRR3332174, SRR3332175, and SRR3332176) were downloaded from the NCBI SRA database [25]. These datasets consisted of 100-bp reads and a library size of 165 bp and contained 28.9, 43.4, and 102.8 million read pairs, respectively. Because the fragment size of these datasets was less than twice the read length, overlapping paired-end reads were merged into long reads using FLASH to obtain 21.2, 28.5, and 81.3 million merged reads, respectively.

We further examined whether SVM filtration could reduce the complexity of the codon-based de Bruijn graph using the ERR188040 dataset. First, the reads were assembled into transcripts using Trinity. Second, two codon-based de Bruijn graphs were constructed using the predicted ORFs of these transcripts before and after SVM filtration, respectively. The node number and the ratio of positive nodes in the components of each graph were calculated.

To assess the performance of inGAP-CDG when identifying orthologous genes for phylogenetic analysis, two 100-bp paired-end RNA-seq datasets were downloaded from the NCBI database (SRA accession numbers of SRR3151756 and SRR2922678). These two datasets contained 45.5 and 45.2 million read pairs and were generated by sequencing brain tissue samples from *H. sapiens* and *M. musculus*, respectively. Because the paired reads in both datasets contained overlaps, we merged these reads into longer reads using FLASH to obtain 27.1 and 26.2 million merged reads, respectively.

### Evaluation of inGAP-CDG performance

Currently, inGAP-CDG is the only tool available that can directly predict genes from unassembled transcriptomic reads. Several tools, such as TransDecoder (http://transdecoder.sourceforge.net/), Prodigal [26], GenMarkS-T [27], and ESTScan [28], predict genes from long transcripts that are generated by transcriptome assemblers (e.g. Trinity, SOAPdenovo-Trans [29], and Oases [30]). Therefore, we built several pipelines to benchmark the performance of inGAP-CDG by combining transcriptome assembly and gene prediction tools. These pipelines include: SOAPdenovo-Trans + ESTScan, SOAPdenovo-Trans + GeneMarkS-T, SOAPdenovo-Trans + Prodigal, SOAPdenovo-Trans + TransDecoder, Trinity + ESTScan, Trinity + GeneMarkS-T, Trinity + Prodigal, Trinity + TransDecoder, Velvet_Oases + ESTScan, Velvet_Oases + Prodigal, and Velvet_Oases + TransDecoder. Furthermore, we prepared a reference gene set to evaluate the performance of these pipelines and inGAP-CDG. We first aligned transcriptomic reads to the hg19 reference genome using TopHat [31] and used Cufflinks [32] to generate transcripts. The resulting transcripts were aligned to the CCDS dataset using BLAT [33] and aligned transcripts with identities greater than 95%, matched lengths longer than 1 kb and coverages greater than 90% were selected as the reference gene set.

For each pipeline and inGAP-CDG, the length distributions of the predicted CDSs were surveyed. The predicted CDSs were aligned to the reference dataset to determine the redundancy, ROC [34], average fragment number per gene, base error rate, and chimera rate. Specifically, alignments with identities greater than 90%, query CDS coverages greater than 90%, and reference gene coverages greater than 90% were reserved. Redundancy was calculated as the number of aligned predicted CDSs divided by the reference gene number. The ROC, which captures the trade-off between sensitivity and specificity, was determined by calculating the true-positive rate (TPR) and the false-positive rate (FPR). Notably, the sensitivity is equal to the TPR, which is the percentage of the reference gene set covered by predicted genes. The specificity is 1-FPR, which represents the percentage of predicted genes covered by the reference gene set. Fragment number, which reflects the number of CDSs belonging to the same reference gene, is computed using the equation $\sum_{i=1}^nn{p}_i$, where $i$ is the number of predicted CDSs aligned to the same reference gene, $n$ is the maximum number of $i$, and ${p}_i$ is the probability of $i$ in all reference genes. The base error rate for a predicted CDS was calculated as the number of mismatches divided by the alignment length. Chimeric CDSs derived from the concatenation of two or more genes were detected and the percentage of chimeras was also calculated. Specifically, the detection strategy was initiated with predicted CDSs larger than 500 bp and these sequences were compared against the reference genes. A predicted CDS was treated as a chimera if it had two or more unique alignments with different genes and each alignment accounted for at least 30% of the length of this CDS.

Moreover, we compared inGAP-CDG with the Trinty + Transdecoder pipeline for orthologous gene recognition using two real RNA-seq datasets from *H. sapiens* and *M. musculus* brain tissue samples. First, CDSs were predicted using these two strategies. inGAP-CDG assembled the transcriptomic reads according to the default parameters. In the pipeline, the reads were assembled into transcripts using Trinity with default parameters and CDSs were predicted by implementing Transdecoder on these transcripts. Next, orthologous gene pairs between *H. sapiens* and *M. musculus* were downloaded from the OrthoMCL database [35]. The one-to-one (one human orthologous gene corresponding to one mouse orthologous gene) orthologous gene pairs were selected and divided into two reference datasets according to their original species. For each species, the predicted proteins obtained from these two strategies were aligned to the reference dataset using BLAT. Finally, the alignments were filtered to identify mapped CDSs. In detail, alignments with identities greater than 95%, query CDS coverages greater than 50%, and reference gene coverages greater than 50% were reserved. If there was only one query CDS uniquely aligned to a reference orthologous gene, the aligned length was greater than 250 codons (i.e. half of the average reference orthologous gene length). For each one-to-one orthologous pair, the numbers of translated proteins aligned to the human and mouse reference orthologous genes were counted, respectively.

## Additional file

Additional file 1:
**Supplementary Method.** Mathematical comparison between the codon-based de Bruijn graph and the traditional de Bruijn graph. **Figure S1.** Efficiency of SVM on removing false positive ORFs. **Figure S2.** Influence of SVM filtration on the assembled CDSs. **Figure S3.** Performance of inGAP-CDG on assembled transcripts. **Figure S4.** Robustness of inGAP-CDG over different sequencing read errors. **Figure S5.** Robustness of inGAP-CDG over different read length. **Figure S6.** Four examples of constructed genes by inGAP-CDG and eight other pipelines. **Figure S7.** Assessment of inGAP-CDG with three real RNA-seq datasets of *D. melanogaster*. **Figure S8.** A detailed pipeline of inGAP-CDG. **Figure S9.** The workflow of six-frame translation. **Figure S10.** Traversing the codon-based de Bruijn graph. **Table S1.** Comparison of the node and edge numbers between traditional de Bruijn graph and codon-based de Bruijn graph. **Table S2.** Datasets used in this study. (PDF 2276 kb)
