# Supplementary material for: A novel codon-based de Bruijn graph algorithm for gene construction from unassembled transcriptomes
Source: Genome Biol. 2016 Nov 17;17:232. doi: 10.1186/s13059-016-1094-x (PMC5114782; doi:10.1186/s13059-016-1094-x)
Supplement: Additional file 1: — Supplementary Method. Mathematical comparison between the codon-based de Bruijn graph and the traditional de Bruijn graph. Figure S1. Efficiency of SVM on removing false positive ORFs. Figure S2. Influence of SVM filtration on the assembled CDSs. Figure S3. Performance of inGAP-CDG on assembled transcripts. Figure S4. Robustness of inGAP-CDG over different sequencing read errors. Figure S5. Robustness of inGAP-CDG over different read length. Figure S6. Four examples of constructed genes by inGAP-CDG and eight other pipelines. Figure S7. Assessment of inGAP-CDG with three real RNA-seq datasets of D. melanogaster. Figure S8. A detailed pipeline of inGAP-CDG. Figure S9. The workflow of six-frame translation. Figure S10. Traversing the codon-based de Bruijn graph. Table S1. Comparison of the node and edge numbers between traditional de Bruijn graph and codon-based de Bruijn graph. Table S2. Datasets used in this study. (PDF 2276 kb) [file 13059_2016_1094_MOESM1_ESM.pdf]

Supplementary Method

Mathematically comparison between the codon-based *de Bruijn* graph and the traditional *de Bruijn* graph

The codon-based *de Bruijn* graph is fundamentally different with the traditional *de Bruijn* graph and has much lower level of complexity and memory usage. In this part, we mathematically prove these advantages of the codon-based *de Bruijn* graph. First, the inputted reads are split into kmers, and for each graph, the number of kmers are:

$$NV(g) = \begin{cases} n(l-k+1), & if\ g = G(V,E) \\ \dfrac{n(l-k+1)}{3}, & if\ g = CG(V,E) \end{cases} \quad (1)$$

Where n is the total number of reads, l is the read length, k is the kmer length, G(V, E) is the traditional *de Bruijn* graph and CG(V, E) is the codon-based *de Bruijn* graph. Considering the repeated kmers, the number of kmers in reality should be less than *NV(g)*. Let A to be a set of kmers and the number of kmers in A is a_n. When a new kmer is added to A, the probability of this kmer is already contained by A is $\frac{a_n}{4^k}$. Therefore, for each graph, the expected kmer number is:

$$NV(g) = \begin{cases} 4^k - (4^k - 1)\left(1 - \dfrac{1}{4^k}\right)^{n(l-k+1)-1}, & if\ g = G(V,E) \\ 4^k - (4^k - 1)\left(1 - \dfrac{1}{4^k}\right)^{\frac{n(l-k+1)}{3}-1}, & if\ g = CG(V,E) \end{cases} \quad (2)$$

It is apparent that the codon-based *de Bruijn* graph contains fewer nodes than the traditional *de Bruijn* graph.

The fraction of branch nodes with out-degree larger than two in the graph largely reflects its complexity. We use E represents the edges which are formed by these branch nodes. For a directed edge,

(i) both head and tail are derived from the same read [13]

For a read, a branch edge appears once with the phenomenon of a repeat node (kmer) in the same read. For n reads, integrating these with the above mentioned (1), (2),

$$E_1 = \begin{cases} n(l-k+1) - [4^k - (4^k - 1)\left(1 - \dfrac{1}{4^k}\right)^{n(l-k+1)-1}], if\ G = G(V,E) \\ \dfrac{n(l-k+1)}{3} - [4^k - (4^k - 1)\left(1 - \dfrac{1}{4^k}\right)^{\frac{n(l-k+1)}{3}-1}], if\ G = CG(V,E) \end{cases}$$

(ii) head and tail are derived from different reads

As we know, the probability of forming an edge is $\frac{1}{4^{k-1}}$ when two kmers in $G(v,e)$, $\frac{1}{4^{k-3}}$ in $CG(v,e)$. Theoretically, for a kmer in a read, the $(n-1)(l-k+1)$ kmers, which are cut by the other $n-1$ reads, can link this kmer to a directed edge ignoring the repeat kmers in

$G(v,e)$, $\frac{(n-1)(l-k+1)}{3}$ in $CG(v,e)$. Because a directed edge is only calculated once when a kmer is linked to the same repeat kmers, we get the $G(e)$ only by computing the number of all kmers cut by the other $n-1$ reads considering the repeat kmers. By integrating these with the above equation (2), for one read,

$$e = \begin{cases} 4^{k-1} - (4^{k-1}-1)\left(1-\frac{1}{4^{k-1}}\right)^{(n-1)(l-k+1)-1} &, if\ G = G(V,E) \\ 4^{k-3} - (4^{k-3}-1)\left(1-\frac{1}{4^{k-3}}\right)^{\frac{(n-1)(l-k+1)}{3}-1} &, if\ G = CG(V,E) \end{cases}$$

for n reads,

$$G(E) = E_1 + ne$$

$$= \begin{cases} n(l-k+1) - [4^k - (4^k-1)\left(1-\frac{1}{4^k}\right)^{n(l-k+1)-1}] + n[4^{k-1} - (4^{k-1}-1)\left(1-\frac{1}{4^{k-1}}\right)^{(n-1)(l-k+1)-1}], if\ G = G(V,E) \\ \frac{n(l-k+1)}{3} - \left[4^k - (4^k-1)\left(1-\frac{1}{4^k}\right)^{\frac{n(l-k+1)}{3}-1}\right] + n[4^{k-3} - (4^{k-3}-1)\left(1-\frac{1}{4^{k-3}}\right)^{\frac{(n-1)(l-k+1)}{3}-1}], if\ G = CG(V,E \end{cases}$$

Set

$$f(x) = \frac{n(l-k+1)}{i} - \left[4^k - (4^k-1)\left(1-\frac{1}{4^k}\right)^{\frac{n(l-k+1)}{x}-1}\right] + n[4^{k-x} - (4^{k-x}-1)\left(1-\frac{1}{4^{k-x}}\right)^{\frac{(n-1)(l-k+1)}{x}-1}]$$

So,

$$f'(x) = -\frac{n(l-k+1)}{x^2} - n(l-k+1)(4^k-1)\ln\left(1-\frac{1}{4^k}\right)\left(1-\frac{1}{4^k}\right)^{\frac{n(l-k+1)}{x}-1}\frac{1}{x^2} - nx4^{k-x}ln4 - n(4^{k-x} - 1)\left(1-\frac{1}{4^{k-x}}\right)^{\frac{(n-1)(l-k+1)}{x}-1}[\frac{1}{4^x-4^k}4^k ln4 + (n-1)(l-k+1)4^x\frac{ln4}{4^x-4^k}\frac{1}{x} - (n-1)(l-k+1)\ln(1-\frac{1}{4^{k-x}})\frac{1}{x^2}]$$

$$f'(3) = -\frac{n(l-k+1)}{9} - \frac{1}{9}n(l-k+1)(4^k-1)\ln\left(1-\frac{1}{4^k}\right)\left(1-\frac{1}{4^k}\right)^{\frac{n(l-k+1)}{3}-1} - \frac{3}{64}n4^k ln4 - n(\frac{4^k}{64} - 1)\left(1-\frac{64}{4^k}\right)^{\frac{(n-1)(l-k+1)}{3}-1}[\frac{1}{64-4^k}4^k ln4 + \frac{64}{3}(n-1)(l-k+1)ln4\frac{1}{64-4^k} - \frac{1}{9}(n-1)(l-k+1)\ln(1-\frac{64}{4^k})]$$

Typically $\ln(1-\frac{64}{4^k}) \approx 0, \ln(1-\frac{1}{4^k}) \approx 0$, and thus,

$$f'(3) = -\frac{n(l-k+1)}{9} - (n-1)(l-k+1)\left(1-\frac{64}{4^k}\right)^{\frac{(n-1)(l-k+1)}{3}-1}$$

$$-\frac{3}{64}n4^k ln4\left[1-\frac{\left(1-\frac{64}{4^k}\right)^{\frac{(n-1)(l-k+1)}{3}-1}}{3}-\frac{1}{9}\frac{(n-1)(l-k+1)}{4^{k-3}}\right]$$

$$\approx -\frac{n(l-k+1)}{9} - (n-1)(l-k+1) - \frac{3}{64}n4^k ln4\left[\frac{2}{3}-\frac{1}{9}\frac{(n-1)(l-k+1)}{4^{k-3}}\right]$$

In practice, $n < 10^8, l \in [50,150]$,

Meanwhile, the k value is set to larger than 20 universally. So, $4^{k-3} > (n-1)(l-k+1)$

So,

$f'(3) \approx -\frac{n(l-k+1)}{9} - (n-1)(l-k+1) - \frac{3}{64}n4^k ln4\left[\frac{2}{3}-\frac{1}{9}\frac{(n-1)(l-k+1)}{4^{k-3}}\right] \approx -\frac{n(l-k+1)}{9} - (n-1)(l-k+1) - \frac{5}{3}n4^{k-3}ln4 < 0$ (4)

Therefore, we conclude that the edge number of the codon-based *de Bruijn* graph is much fewer than that of the traditional *de Bruijn* graph. Owing to its small number of nodes and edges, the codon-based *de Bruijn* graph saves traversing time and memory usage.

Figure S1. **Efficiency of SVM on removing false positive ORFs**. (**A**) The ROC curve of simulated RNA-seq datasets from chr3 of the human genome (hg19) after SVM filtration. (**B**) The ROC curve of simulated RNA-seq datasets from chr19 of the human genome (hg19) after SVM filtration. (**C**) The ROC curve of simulated RNA-seq datasets from chr20 of the human genome (hg19) after SVM filtration. (**D**) The ratio of positive and negative kmers in the simplified graph after tips trimming and bubbles merging on simulated RNA-seq datasets from chr3 of the human genome (hg19). (**E**) The ratio of positive and negative kmers in the simplified graph after tips trimming and bubbles merging on simulated RNA-seq datasets from chr19 of the human genome (hg19). (**F**) The rate of positive and negative kmers in the simplified graph after tips trimming and bubbles merging on simulated RNA-seq datasets from chr20 of the human genome (hg19).

Figure S2. **Influence of SVM filtration on the assembled CDSs**. inGAP-CDG predicted CDSs from a real RNA-seq dataset (SRR1045067) with or without SVM filtration in the algorithm. (**A**) The length distribution of predicted CDSs. (**B**) The mean, N50 and N90 length of predicted CDSs. (**C**) Comparison on the redundancy of predicted CDSs. (**D**) Comparison on the ROC curve. (**E**) Comparison on the gene fragmentation between the two approaches.

Figure S3. **Performance of inGAP-CDG on assembled transcripts**. The RNA-seq dataset (SRR1045067) was first assembled using Trinity, and the assembled long transcripts were then used for gene prediction by inGAP-CDG and TransDecoder, respectively. (**A**) The length distribution of the two methods. (**B**) The mean, N50 and N90 length of the two methods. (**C**) Comparison on the redundancy of predicted CDSs. (**D**) Comparison on the ROC curve. (**E**) Comparison on the gene fragmentation between the two methods.

Figure S4. **Robustness of inGAP-CDG over different sequencing read errors**. inGAP-CDG and eleven other pipelines predicted CDSs from three simulated RNA-seq dataset with different sequencing read errors read (0.5%, 1% and 2%), respectively. (**A**) Comparison on the redundancy of predicted CDSs. (**B**) Comparison on the sensitivity CDS prediction. (**C**) Comparison on the mean length of predicted CDSs. (**D**) Comparison on the error rate of predicted CDSs.

Figure S5. **Robustness of inGAP-CDG over different read length**. inGAP-CDG and eleven other pipelines predicted CDSs from three real RNA-seq datasets (ERR188040, ERR1161592, and SRR1045067) with different read length (75, 100 and 150 bp), respectively. (**A**) Comparison on the mean length of predicted CDSs. (**B**) Comparison on the sensitivity and specificity of true CDS prediction. (**C**) Comparison on the gene fragmentation among the nine methods. (**D**) Comparison on the redundancy of predicted CDSs.

Figure S6. **Four examples of constructed genes by inGAP-CDG and eight other pipelines**. Four human genes downloaded from NCBI were used as the reference genes, the predicted CDSs by inGAP-CDG and eight other pipelines were aligned to their reference genes, respectively. Black lines indicate the aligned fragments in the predicted CDSs. Green lines represent the unaligned fragments in the predicted CDSs. Notably, the pipelines using Trinity or Velvet as transcriptome assembler yielded a large number of fragmented and redundant CDS predictions. (**A**) XM_011533632. (**B**) NM_004168. (**C**) NM_013379. (**D**) NM_000018.

Figure S7. **Assessment of inGAP-CDG with three real RNA-seq data sets of *Drosophila melanogaster***. inGAP-CDG and eleven other pipelines predicted CDSs from three real RNA-

seq data sets (SRR3331274, ERR3331275 and SRR3331276), respectively. Four metrics (mean length, sensitivity, redundancy and chimera rate) were used to assess the performance of inGAP-CDG and eleven other pipelines.

Figure S8. **A detailed pipeline of inGAP-CDG**.

Figure S9. **The workflow of six-frame translation**. The translated ORFs are sorted by length. Predicted ORFs were filtered following two different criteria (loose and strict).

Figure S10. **Traversing the codon-based *de Bruijn* graph**. (**A, B**) Tips in the codon-based *de Bruijn* graph. Tips are trimmed iteratively if their length <= 2*kmer. (**C, D**) Bubbles in the codon-base *de Bruijn* graph. The paths in a bubble are compared and their pairwise identity is calculated. If the identity reaches a cutoff of 95%, the longest path is reserved and all other paths are discarded. (**E**) rORFs can be served as landmarks for graph traversal. (**F**) A full-length CDS will be outputted if the CDS path is supported by the rORFs.

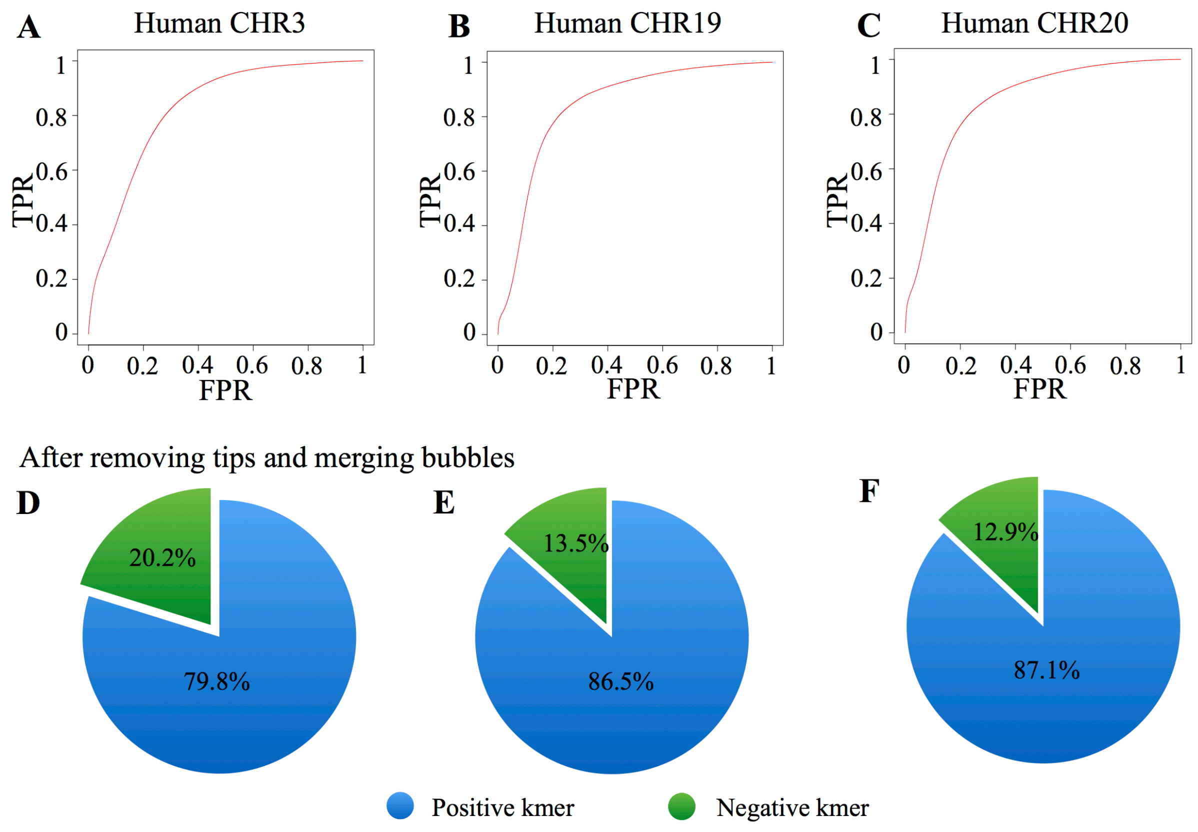
A
Human CHR3
B
Human CHR19
C
Human CHR20
TPR
FPR
0
0.2
0.4
0.6
0.8
1
After removing tips and merging bubbles
D
20.2%
79.8%
E
13.5%
86.5%
F
12.9%
87.1%
Positive kmer
Negative kmer

Figure S1

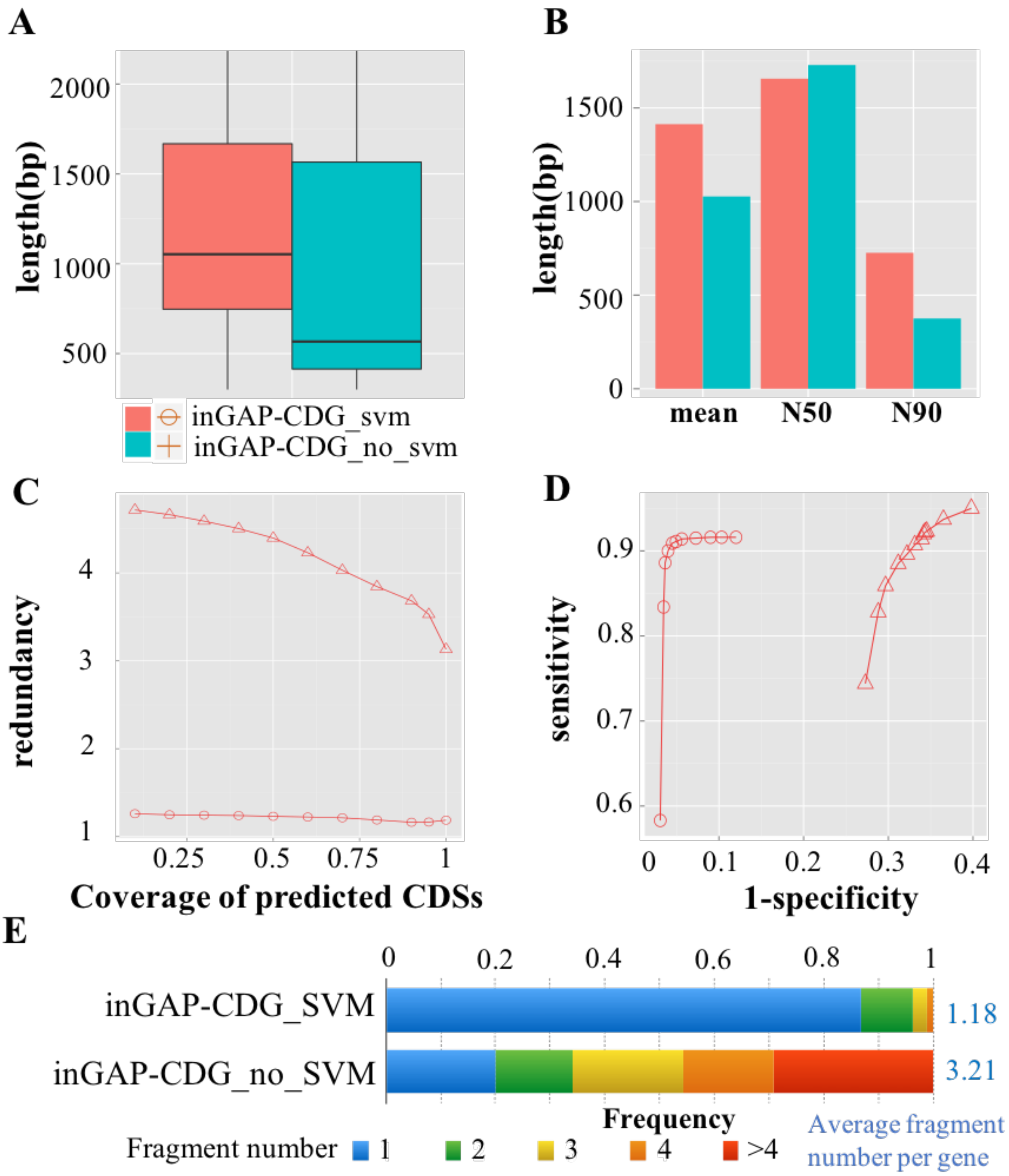
A
B
C
D
E
length(bp)
2000
1500
1000
500
0
mean
N50
N90
inGAP-CDG_svm
inGAP-CDG_no_svm
redundancy
0.25
0.5
0.75
1
Coverage of predicted CDSs
sensitivity
0.9
0.8
0.7
0.6
0.1
0.2
0.3
0.4
1-specificity
inGAP-CDG_SVM
inGAP-CDG_no_SVM
1.18
3.21
Frequency
Fragment number
>4
Average fragment number per gene

Figure S2

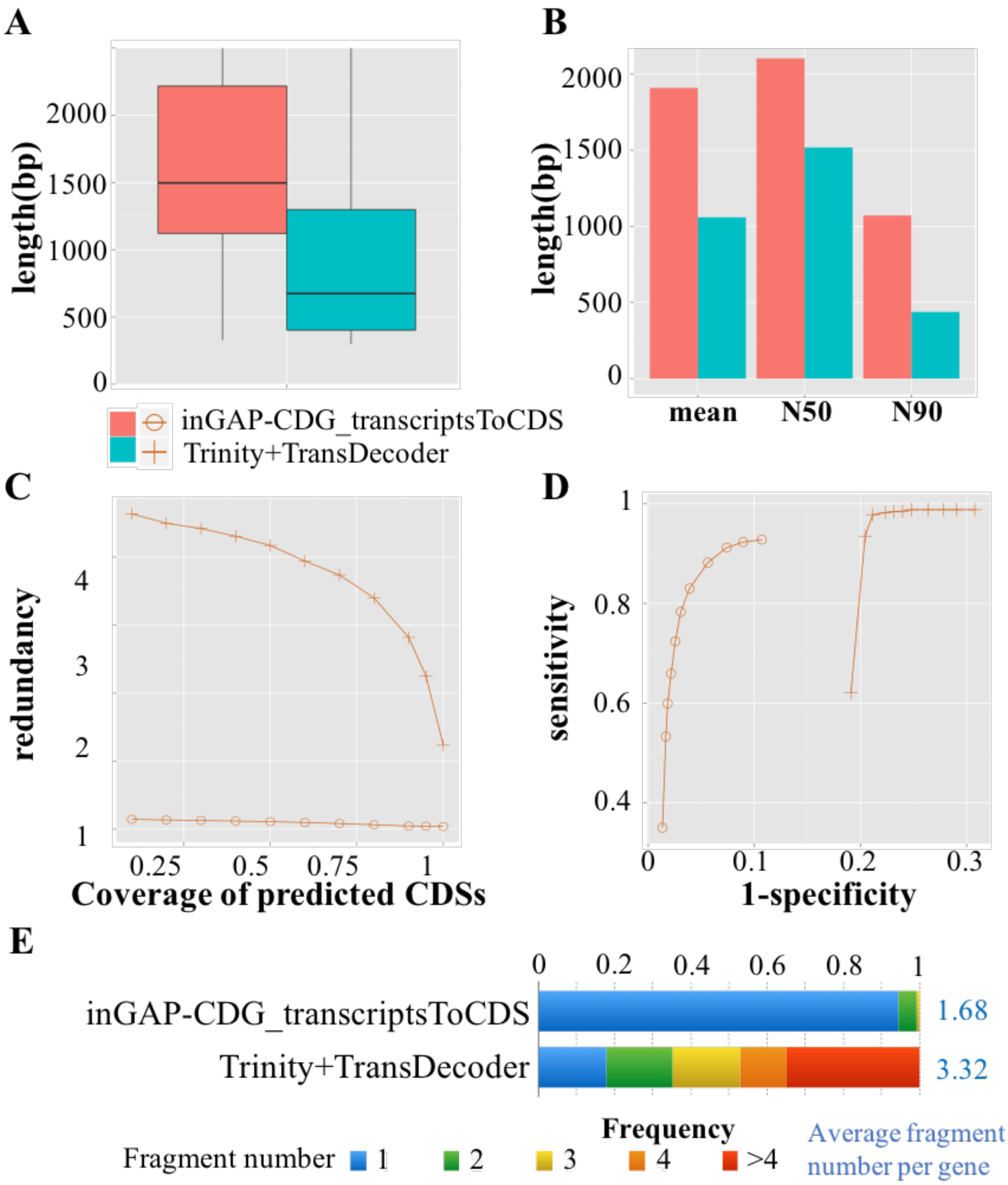

A
length(bp)
2000
1500
1000
500
0
B
length(bp)
2000
1500
1000
500
0
mean
N50
N90
inGAP-CDG_transcriptsToCDS
Trinity+TransDecoder
C
redundancy
4
3
2
1
0.25
0.5
0.75
1
Coverage of predicted CDSs
D
sensitivity
1
0.8
0.6
0.4
0
0.1
0.2
0.3
1-specificity
E
0
0.2
0.4
0.6
0.8
1
inGAP-CDG_transcriptsToCDS
Trinity+TransDecoder
1.68
3.32
Frequency
Fragment number 1 2 3 4 >4
Average fragment number per gene

Figure S3

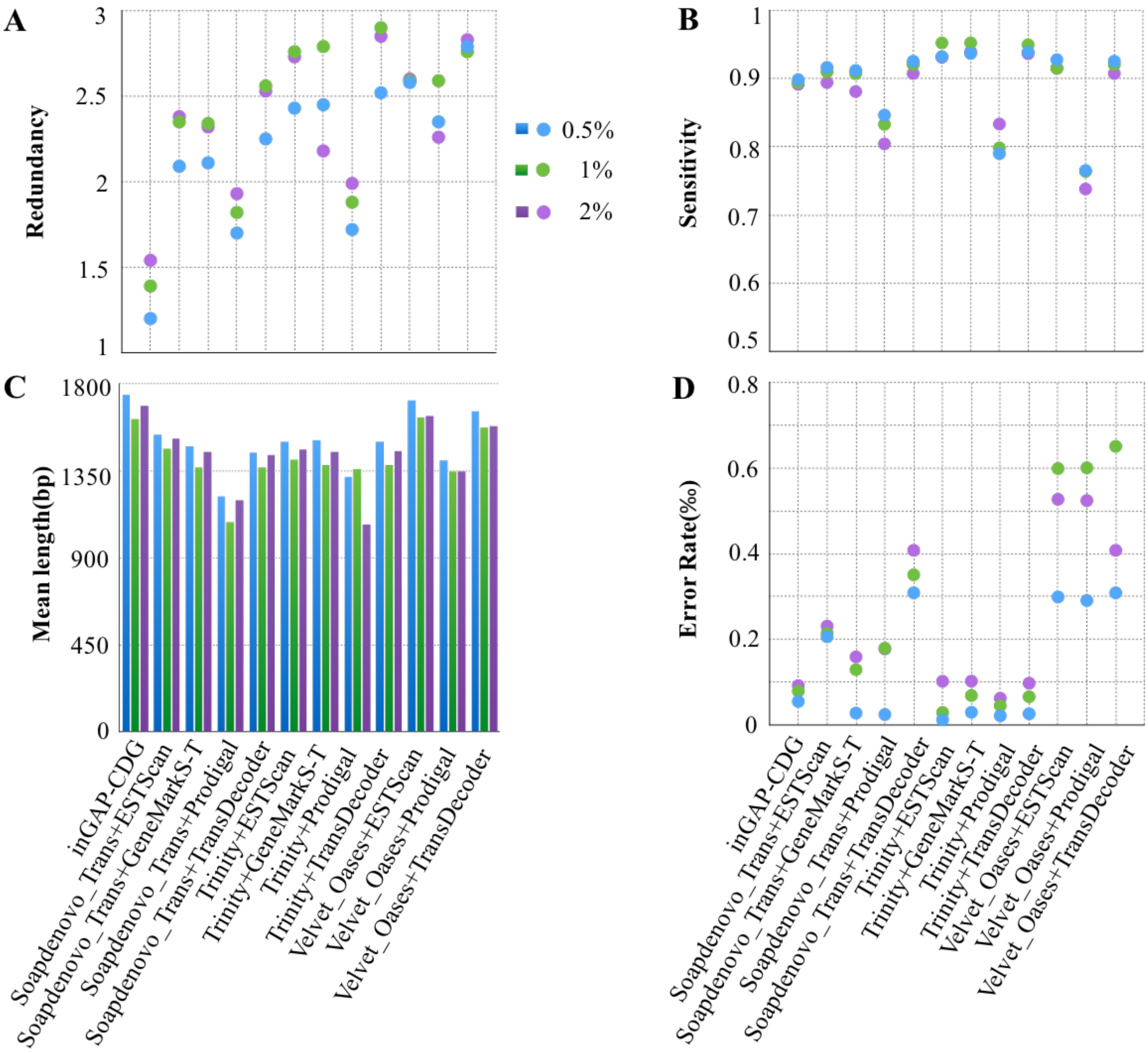

A
Redundancy
B
Sensitivity
C
Mean length(bp)
D
Error Rate(%)
0.5%
1%
2%
inGAP-CDG
Soapdenovo_Trans+ESTScan
Soapdenovo_Trans+GeneMarkS-T
Soapdenovo_Trans+Prodigal
Soapdenovo_Trans+TransDecoder
Trinity+ESTScan
Trinity+GeneMarkS-T
Trinity+Prodigal
Trinity+TransDecoder
Velvet_Oases+ESTScan
Velvet_Oases+Prodigal
Velvet_Oases+TransDecoder

Figure S4

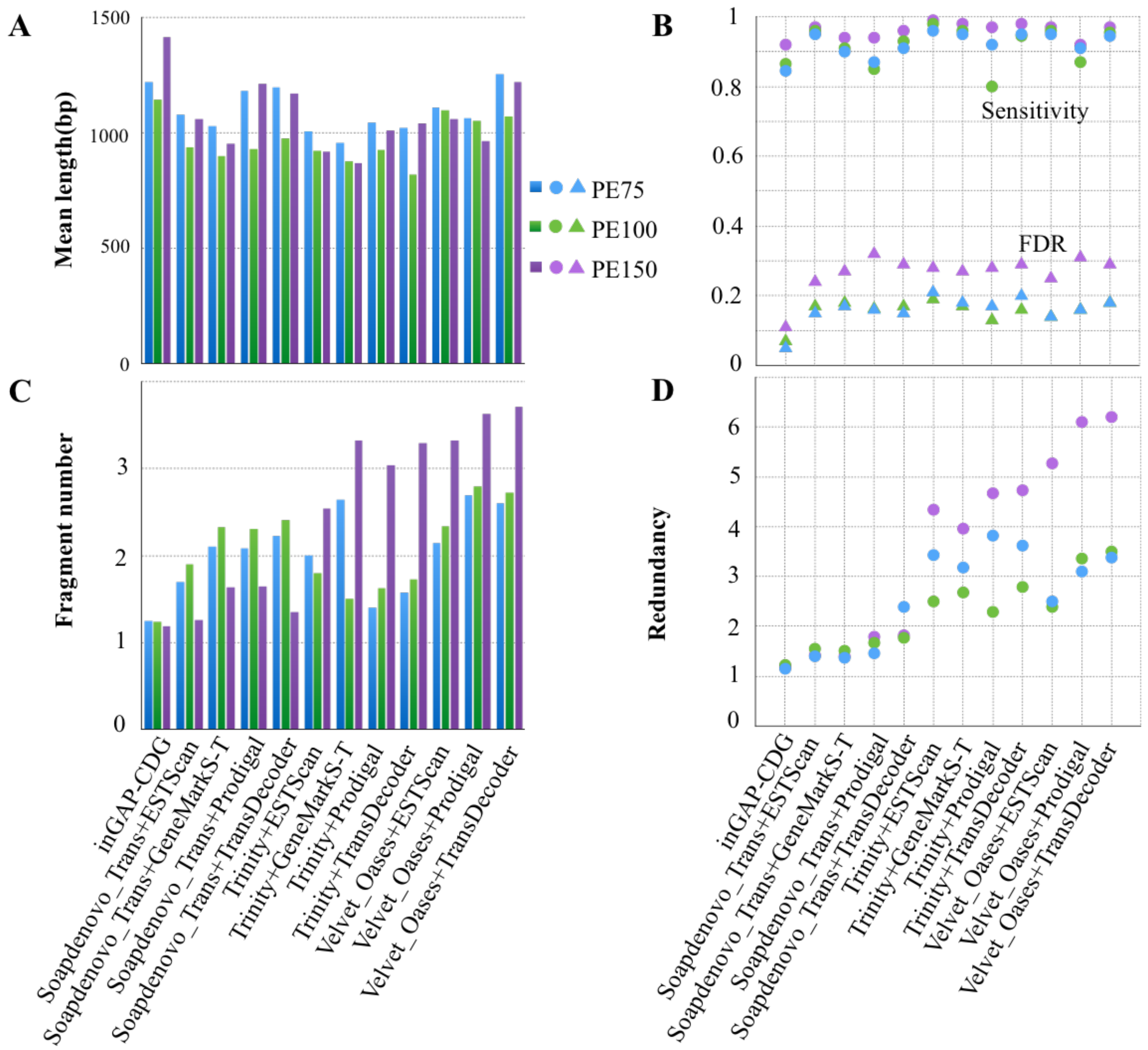
A
B
C
D
1500
1000
500
0
Mean length(bp)
PE75
PE100
PE150
1
0.8
0.6
0.4
0.2
0
Sensitivity
FDR
Fragment number
3
2
1
0
6
5
4
3
2
1
0
Redundancy
inGAP-CDG
Soapdenovo_Trans+ESTScan
Soapdenovo_Trans+GeneMarkS-T
Soapdenovo_Trans+Prodigal
Soapdenovo_Trans+TransDecoder
Trinity+ESTScan
Trinity+GeneMarkS-T
Trinity+Prodigal
Trinity+TransDecoder
Velvet_Oases+ESTScan
Velvet_Oases+Prodigal
Velvet_Oases+TransDecoder

Figure S5

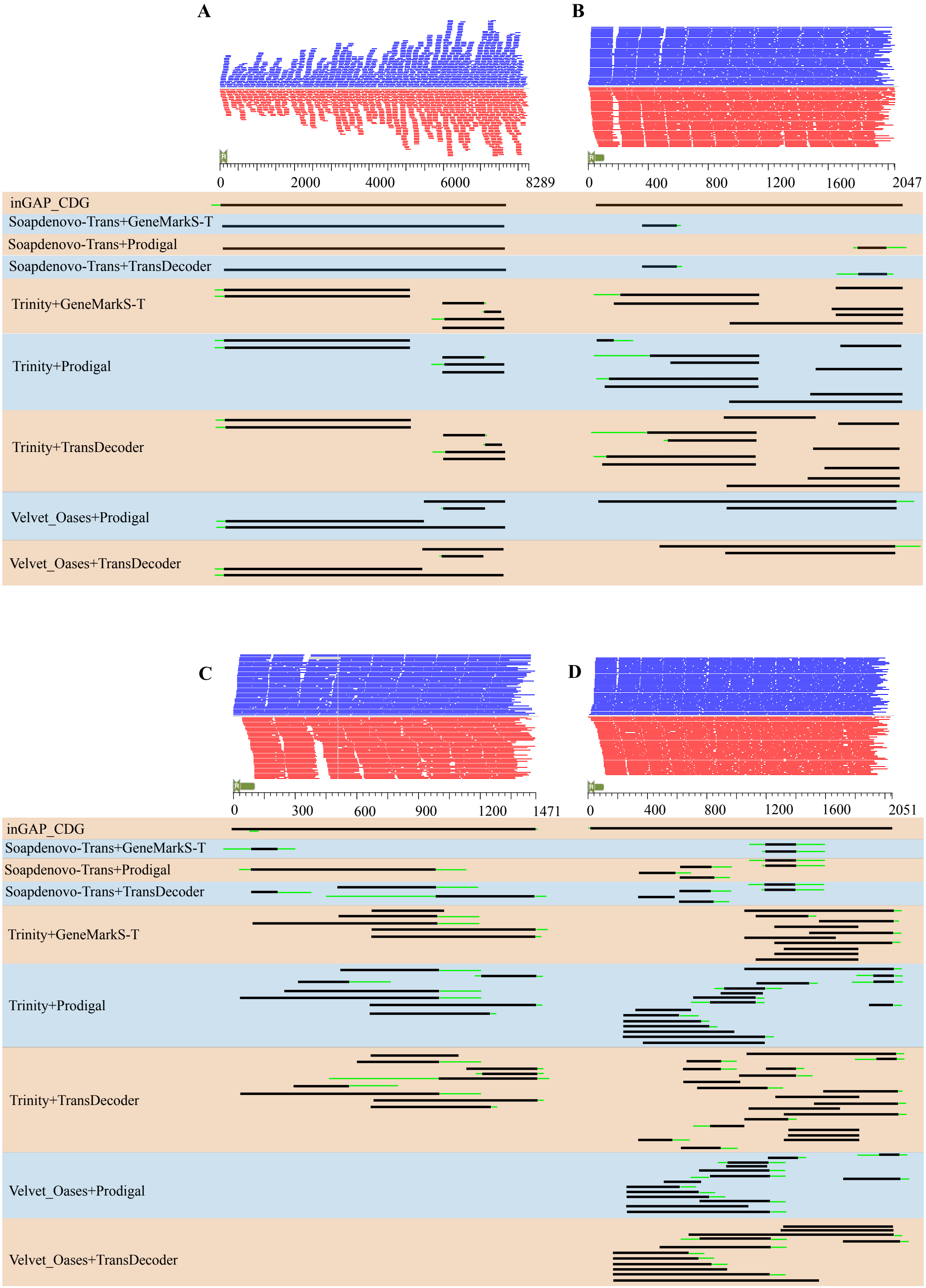
A
B
0
2000
4000
6000
8289
0
400
800
1200
1600
2047
inGAP_CDG
Soapdenovo-Trans+GeneMarkS-T
Soapdenovo-Trans+Prodigal
Soapdenovo-Trans+TransDecoder
Trinity+GeneMarkS-T
Trinity+Prodigal
Trinity+TransDecoder
Velvet_Oases+Prodigal
Velvet_Oases+TransDecoder
C
D
0
300
600
900
1200
1471
0
400
800
1200
1600
2051
inGAP_CDG
Soapdenovo-Trans+GeneMarkS-T
Soapdenovo-Trans+Prodigal
Soapdenovo-Trans+TransDecoder
Trinity+GeneMarkS-T
Trinity+Prodigal
Trinity+TransDecoder
Velvet_Oases+Prodigal
Velvet_Oases+TransDecoder

Figure S6

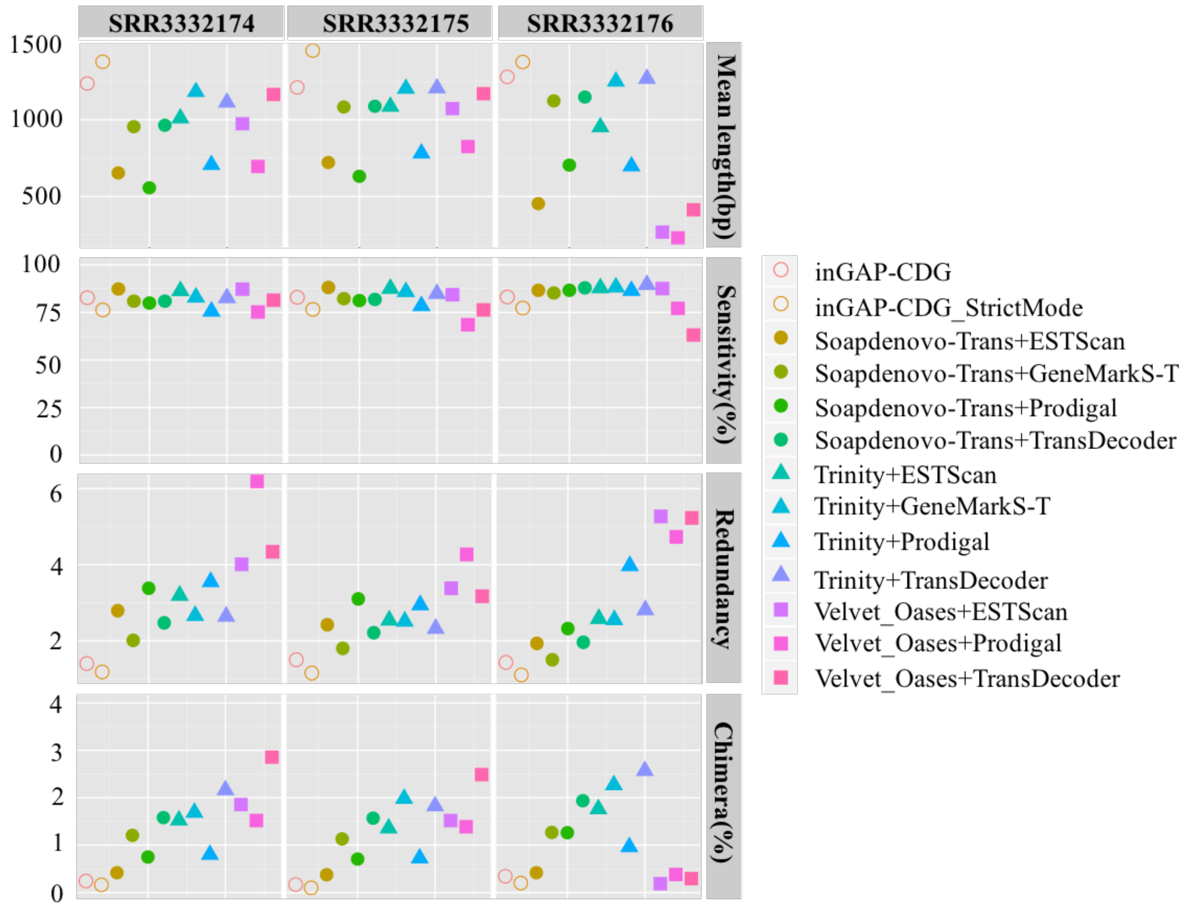

SRR3332174
SRR3332175
SRR3332176
1500
1000
500
Mean length(bp)
100
75
50
25
0
Sensitivity(%)
6
4
2
Redundancy
4
3
2
1
0
Chimera(%)
inGAP-CDG
inGAP-CDG_StrictMode
Soapdenovo-Trans+ESTScan
Soapdenovo-Trans+GeneMarkS-T
Soapdenovo-Trans+Prodigal
Soapdenovo-Trans+TransDecoder
Trinity+ESTScan
Trinity+GeneMarkS-T
Trinity+Prodigal
Trinity+TransDecoder
Velvet_Oases+ESTScan
Velvet_Oases+Prodigal
Velvet_Oases+TransDecoder

Figure S7

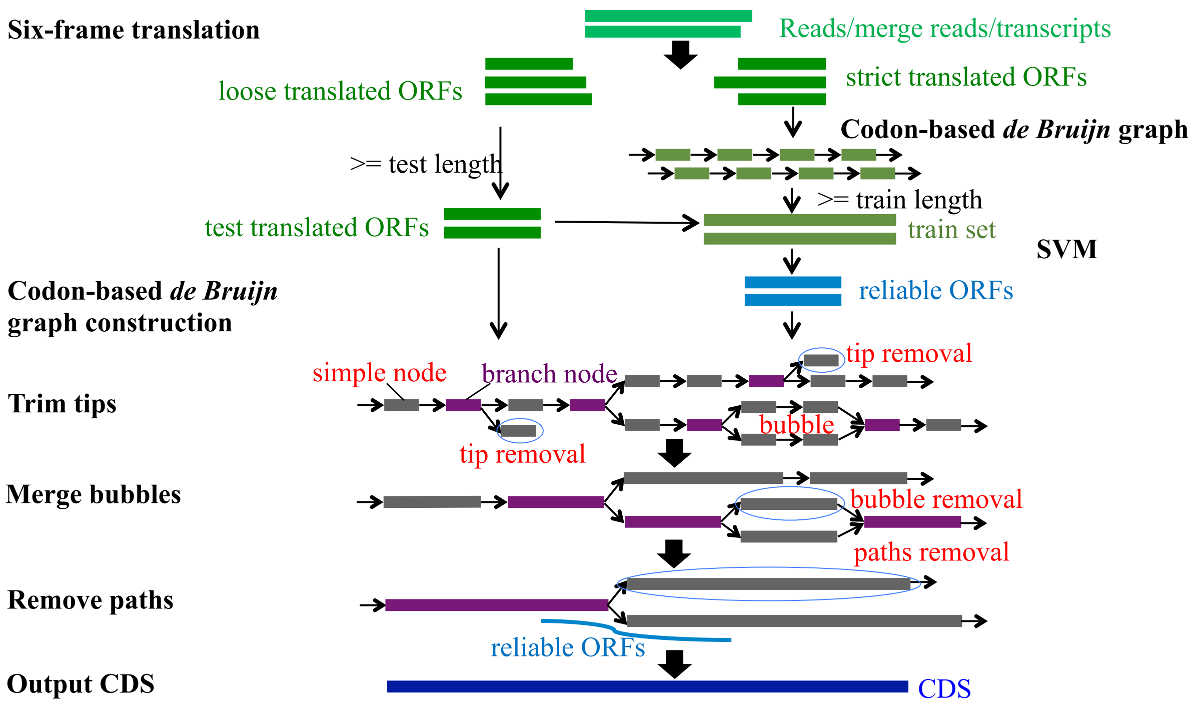
Six-frame translation
Reads/merge reads/transcripts
loose translated ORFs
strict translated ORFs
Codon-based *de Bruijn* graph
>= test length
>= train length
test translated ORFs
train set
SVM
Codon-based *de Bruijn* graph construction
reliable ORFs
simple node
branch node
tip removal
Trim tips
bubble
tip removal
Merge bubbles
bubble removal
paths removal
Remove paths
reliable ORFs
Output CDS
CDS

Figure S8

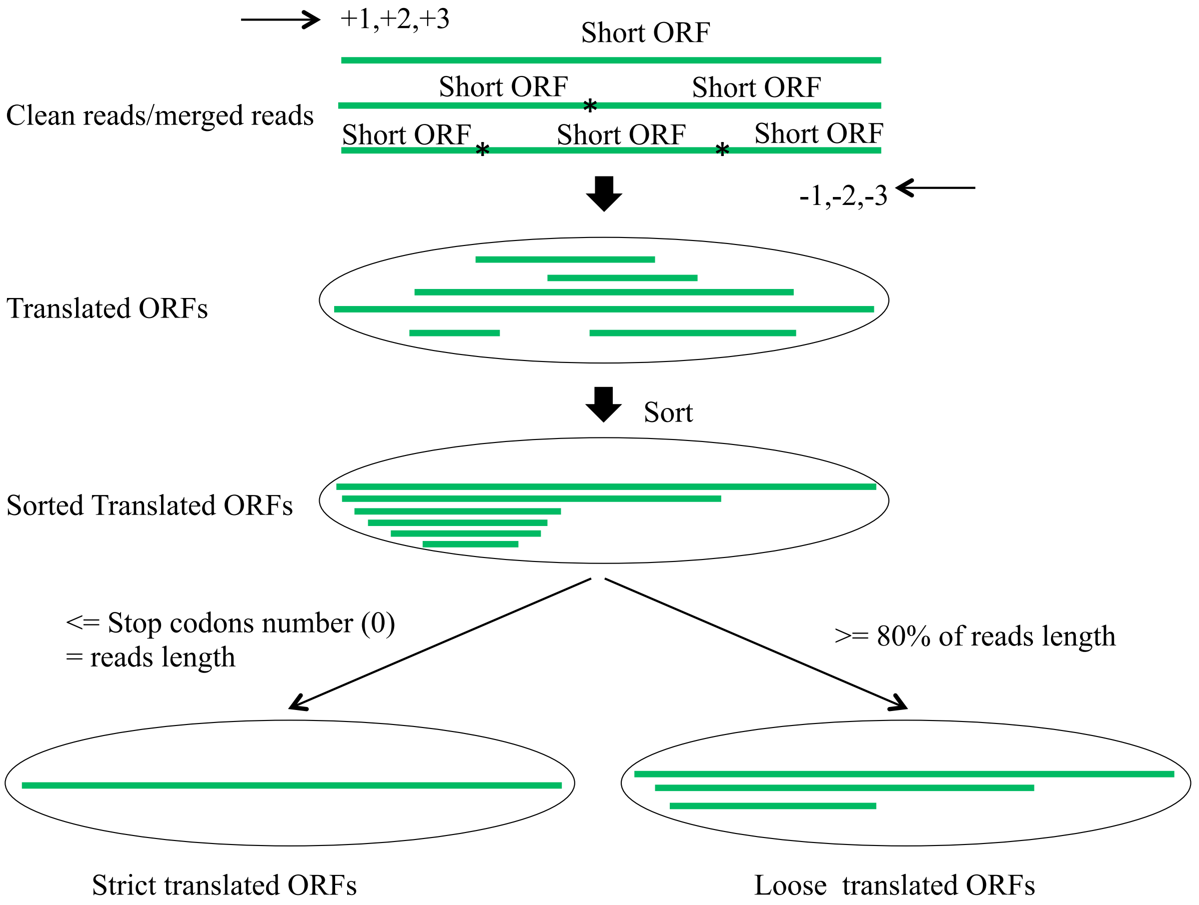

+1,+2,+3
Short ORF
Short ORF
Short ORF
Clean reads/merged reads
Short ORF
Short ORF
Short ORF
-1,-2,-3
Translated ORFs
Sort
Sorted Translated ORFs
<= Stop codons number (0)
= reads length
>= 80% of reads length
Strict translated ORFs
Loose translated ORFs

Figure S9

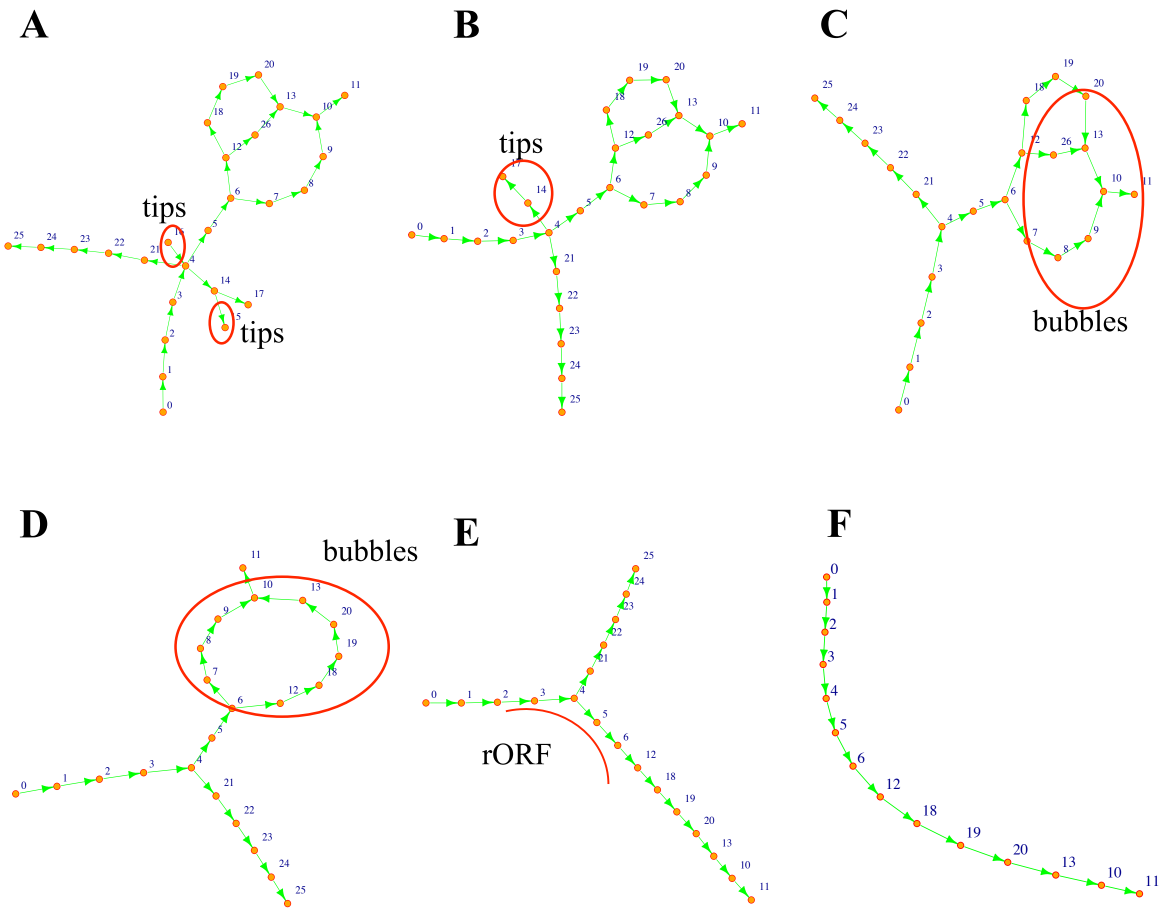

Figure S10

Table S1. **Comparison of the node and edge numbers between traditional *de Bruijn* graph and codon-based *de Bruijn* graph.**

	Data		Node number		Edge number	
	size(M)	number	k-1	k-3	k-1	k-3
human ccds	48	28,708	31,310,227	10,448,659	31,324,270	10,474,630
mouse ccds	37	23,089	31,489,646	10,493,453	31,503,979	10,518,294
fruit fly ccds	66	29,375	22,264,470	7,425,159	22,263,472	7,433,273
ERR188040_1.clean.fastq	3,200	24,025,738	181,805,822	94,908,406	182,169,581	96,359,260
ERR1161592_1.clean.fastq	3,500	20,996,217	201,359,022	96,633,034	202,425,673	99,713,421
SRR1045067_1.clean.fastq	2,900	16,267,330	286,703,917	150,956,431	289,682,788	158,070,462

Table S2. **Datasets used in this study.**

Species	Sequence number	Description
Homo sapiens	28,708	CDS
Homo sapiens (chr3)	1,629	CDS
Homo sapiens (chr3)	1,685,395	100bp, single-end reads
Homo sapiens (chr3)	1,477,272	300bp, single-end reads
Homo sapiens (chr3)	1,277,811	500bp, single-end reads
Homo sapiens (chr3)	1,012,378	800bp, single-end reads
Homo sapiens (chr3)	2*1,500,000	2*200bp, simulated paired-end reads, 0.5% sequenced error
Homo sapiens (chr3)	2*1,500,000	2*200bp, simulated paired-end reads, 1% sequenced error
Homo sapiens (chr3)	2*1,500,000	2*200bp, simulated paired-end reads, 2% sequenced error
Homo sapiens (chr19)	1,936	CDS
Homo sapiens (chr20)	787	CDS
Homo sapiens (ERR188040)	2*27,256,165	2* 75bp, paired-end reads
Homo sapiens (ERR1161592)	2*24,418,242	2*100bp, paired-end reads
Homo sapiens (SRR1045067)	2*19,169,171	2*150bp, paired-end reads
Homo sapiens (SRR3151756)	2*45,497,333	2*100bp, paired-end reads
Homo sapiens (SRR2922678)	2*45,199,432	2*100bp, paired-end reads
Drosophila melanogaster (SRR3332174)	2*28,858,597	2*100bp, paired-end reads
Drosophila melanogaster (SRR3332175)	2*43,304,279	2*100bp, paired-end reads
Drosophila melanogaster (SRR3332176)	2*102,788,015	2*100bp, paired-end reads
